# Pyrroloquinoline Quinone Modifies Lipid Profile, but Not Insulin Sensitivity, of Palmitic Acid-Treated L6 Myotubes

**DOI:** 10.3390/ijms21218382

**Published:** 2020-11-08

**Authors:** Elżbieta Supruniuk, Agnieszka Mikłosz, Adrian Chabowski

**Affiliations:** Department of Physiology, Medical University of Bialystok, Mickiewicza 2C Street, 15-222 Bialystok, Poland; agnieszka.miklosz@umb.edu.pl (A.M.); adrian@umb.edu.pl (A.C.)

**Keywords:** pyrroloquinoline quinone, PGC-1α, lipid metabolism, insulin sensitivity, L6 myotubes

## Abstract

Pyrroloquinoline quinone (PQQ) is a novel stimulator of mitochondrial biogenesis and cellular energy metabolism. This is the first study investigating regulatory mechanisms and metabolic responses underlying PQQ’s action in palmitate-exposed L6 myotubes. Particularly, we assessed alterations in lipid content and composition, expression of metabolic enzymes, and changes in glucose transport. The experiments were conducted using muscle cells subjected to short (2 h) and prolonged (24 h) incubation with PQQ in a sequence of pre- and post-palmitic acid (PA) exposure. We demonstrated the opposite effects of 2 and 24 h treatments with PQQ on lipid content, i.e., a decline in the level of free fatty acids and triacylglycerols in response to short-time PQQ incubation as compared to increases in diacylglycerol and triacylglycerol levels observed after 24 h. We did not demonstrate a significant impact of PQQ on fatty acid transport. The analysis of metabolic enzyme expression showed that the vast majority of PQQ-dependent alterations cumulated in the PA/PQQ 24 h group, including elevated protein amount of peroxisome proliferator activated receptor γ co-activator 1α (PGC-1α), sirtuin-1 (SIRT1), phosphorylated 5′AMP-activated protein kinase (pAMPK), carnitine palmitoyltransferase I (CPT1), citrate synthase (CS), fatty acid synthase (FAS), and serine palmitoyltransferase, long chain base subunit 1 (SPT1). In conclusion, the results mentioned above indicate PQQ-dependent activation of both fatty acid oxidation and lipid synthesis in order to adapt cells to palmitic acid-rich medium, although PQQ did not attenuate insulin resistance in muscle cells.

## 1. Introduction

The sequential development of tissue-specific insulin resistance and metabolic disruptions, rather than simultaneous whole-body desensitization to insulin, serves as the preferential pathological background for type 2 diabetes [1]. Skeletal muscles are particularly essential in maintaining energy balance due to their high metabolic activity and large contribution to postprandial insulin-stimulated glucose clearance (80–90%). In this regard, muscle cells are a consequential cell type for lipid homeostasis disorders [2]. The importance of reducing peripheral lipids storage can be supported by the results obtained with ^1^H magnetic resonance spectroscopy (^1^H MRS) in humans, revealing an association between intramyocellular lipids (IMCL) accumulation and insulin resistance independent of total fat mass [3,4].

Excessive deposition of deleterious fatty acid metabolites in non-adipose tissues intimately contributes to the development of insulin resistance. It is now widely recognized that a protein-mediated process is engaged in the transport of long-chain fatty acids (LCFAs) across plasma membrane. So far, a number of fatty acid transport proteins have been identified, including cluster of differentiation 36/scavenger receptor class B protein (CD36/SR-B2), plasma membrane-associated fatty acid binding proteins (FABPpm), and a family of fatty acid transport proteins (FATP1-6). Trafficking of these proteins from an intracellular depot to the plasma membrane is connected with the dysregulation of fatty acid metabolism in skeletal muscle [5]. This refers to inevitable overwhelming of mitochondrial β-oxidation capacity and the formation of lipotoxic fractions, such as diacylglycerols (DAG) and ceramides (CER). Recent studies have revealed that abnormal DAG content contributed to insulin resistance in different animal models [6,7] due to the activation of conventional protein kinase C (PKC) (α, βI, βII, γ) and novel PKC (δ, ε, η, θ) isoforms that inhibited insulin receptor substrate 1 (IRS1) phosphorylation [8,9]. In turn, CER antagonize insulin signaling via activation of protein phosphatase 2A (PP2A) and protein kinase C λ/ζ (PKCλ/ζ), both of which are implicated in the dephosphorylation and reduction of protein kinase B/Akt activity (a molecule and central hub of insulin signaling pathway). Additionally, CER can directly stimulate IκB kinase (IKK) and c-Jun N-terminal kinase (JNK), that prevent IRS1 phosphorylation and eventually inhibit insulin transmission pathway [10,11]. Finally, triacylglycerols (TAG) appear to be relatively benign with respect to the impairment of insulin signaling since neutral lipid deposition is often present in skeletal muscle of endurance trained athletes [12]. In this regard, relieving insulin resistance through the modulation of lipid metabolism is constantly investigated.

Pyrroloquinoline quinone (PQQ) disodium salt is an anionic water-soluble, heat-stable compound initially discovered as a redox cofactor for alcohol and glucose dehydrogenases in methylotrophic bacteria [13]. It is ubiquitously detected at pico- to nanomolar concentrations in mammalian tissues, human milk and in 5–10 times higher amounts in multiple dietary sources (i.e., vegetables, fruits, teas) [14,15], while the predicted everyday dietary dose for humans ranges from 0.1 to 1 mg of PQQ and its derivatives [16]. PQQ is a multifunctional bio-agent exerting anti-inflammatory [16,17,18], growth-promoting [19,20], anti-oxidative [21], anti-cancer and anti-aging effects [22]. PQQ has been shown to affect a wide range of genes, especially those involved in mitochondria-related functions. Importantly, due to redox activity, PQQ can oxidize reduced nicotinamide adenine dinucleotide (NADH) to generate NAD^+^ and enhance the NAD^+^ dependent metabolic response in cells [23]. Most of the established PQQ effects have been associated with the stimulation of peroxisome proliferator activated receptor γ co-activator 1α (PGC-1α) through the phosphorylation of cyclic AMP response element binding protein (CREB) at serine 133 [24,25]. Transcriptional regulation of PGC-1α target genes involves the co-activation of transcription factor-mediated gene expression as well as modulation of alternative splicing of the nascent transcript [26]. The regulatory effects of PGC-1α in the pathogenesis of insulin resistance and type 2 diabetes have been manifested in different animal models, and associated with the modulation of multiple genes controlling lipolysis and lipogenesis [27]. Likewise, PQQ supplementation increased metabolic flexibility in obese mice offspring, simultaneously shifting liver lipid composition towards increased TAG content with a decline in the lipotoxic pools, i.e., CER and sphingomyelin [28].

To date, it is not clear whether PQQ is capable of attenuating insulin resistance in muscle cells. Previously, we evaluated dose- and time-dependent differences in the influence of PQQ on lipid metabolism in L6 skeletal muscle cells in basal conditions. We reported the role of PQQ and PGC-1α in the esterification of fatty acids to specific lipid pools, such as TAG and CER, i.e., protective and deleterious fractions, respectively, for the appropriate cellular function [29]. The impact of short- (2 h) and long-term (24 h) exposure to PQQ on particular lipid fraction content and composition as well as the expression of enzymes involved in lipid metabolism in insulin-resistant L6 myotubes is described herein. Our analysis encompasses enzymes involved in fatty acids transport (i.e., CD36/SR-B2, FABPpm, FATP1, FATP4), β-oxidation (i.e., PGC-1α; sirtuin-1, SIRT1; peroxisome proliferator activated receptor γ, PPARγ; 5′AMP-activated protein kinase, AMPK; phosphorylated 5′AMP-activated protein kinase, pAMPK; 3-hydroxyacyl-CoA dehydrogenase, β-HAD; carnitine palmitoyltransferase I, CPT1; citrate synthase, CS), TAG and DAG metabolism (i.e., diacylglycerol O-acyltransferase 1, 2, DGAT1 and DGAT2; adipose triglyceride lipase, ATGL), de novo lipogenesis (i.e., fatty acid synthase, FAS) and ceramide formation (i.e., serine palmitoyltransferase, long chain base subunit 1, SPT1). Moreover, we examined the impact of PQQ on insulin responsiveness (i.e., Akt and IRS1 phosphorylation, glucose transporter type 4 (GLUT4) expression, glucose transport) in the above-mentioned model.

## 2. Results

### 2.1. The Impact of Pre- and Post-Incubation with PQQ on the Content and Composition of Selected Lipid Fractions in Insulin-Resistant L6 Myotubes

#### 2.1.1. Free Fatty Acid (FFA) Content and Composition in L6 Myotubes

**Figure 1:**
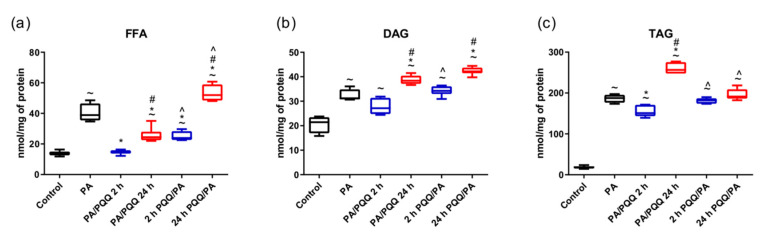
Effects of short- and long-time incubation with pyrroloquinoline quinone (PQQ) on the intracellular content of (**a**) free fatty acids, (**b**) diacylglycerols, and (**c**) triacylglycerols in L6 myotubes. The values are presented as mean ± SEM and are based on six independent determinations. The Kruskal–Wallis test followed by post hoc adjustment was applied. ^~^ *p* < 0.05, study groups versus control; * *p* < 0.05, PQQ-incubated groups versus palmitate group; ^#^ *p* < 0.05, time-dependent differences (long- vs. short-time incubation with PQQ); ^ *p* < 0.05, sequence-dependent differences (pre-incubation with PQQ vs. post-incubation with PQQ). ANOVA: analysis of variance; PA: palmitic acid; PQQ: pyrroloquinoline quinone.

We demonstrated that palmitic acid treatment significantly elevated total FFA content in muscle cells because of an increase in saturated and unsaturated fatty acids (+177%, +194%, +33%, palmitic acid (PA) vs. control, respectively, *p* < 0.05; Figure 1a, Table A1).

Distinct differences in lipid content and composition were observed in PQQ treated L6 myotubes. First, we ascertained whether the dose of 0.5 µM PQQ could modify the effects of palmitate exposure on FFA level. Post-incubation of muscle cells with PQQ decreased the content of total FFA and saturated fatty acids as compared to the palmitate group (total: −62%, −37%; UNSAT: −66%, −41%, 2 h and 24 h of PA/PQQ vs. PA, respectively, *p* < 0.05; Figure 1a, Table A1). Additionally, time-dependent differences were demonstrated for the total FFA level as well as the amount of saturated and unsaturated fatty acids (+67%, +75%, +23%, 24 h vs. 2 h of PA/PQQ, respectively, *p* < 0.05; Figure 1a, Table A1).

The presented data also revealed that short-term pre-incubation with 0.5 μM of PQQ substantially diminished FFA level in L6 cells concomitantly with reduced concentration of saturated fatty acid species as compared to the palmitate group (−39%, −40%, 2 h PQQ/PA vs. PA, respectively, *p* < 0.05). On the contrary, the amount of FFA and their saturated species increased after 24 h pre-treatment with PQQ (+34%, +35%, 24 h PQQ/PA vs. PA, respectively, *p* < 0.05). Time-dependent differences were noticed for total FFA and saturated fatty acid content (+118%, +124%, 24 h vs. 2 h of PQQ/PA, respectively, *p* < 0.05; Figure 1a, Table A1).

Moreover, we noticed significant differences in the effects of pre- and post-incubation with PQQ in both periods of PQQ administration. These changes encompassed an increase in total FFA (+63%, +113%, 2 h PQQ/PA vs. PA/PQQ 2 h, 24 h PQQ/PA vs. PA/PQQ 24 h, respectively, *p* < 0.05) and saturated fatty acid content (+77%, +127%, 2 h PQQ/PA vs. PA/PQQ 2 h, 24 h PQQ/PA vs. PA/PQQ 24 h, respectively, *p* < 0.05; Figure 1a, Table A1) in pre-incubated cells as compared to post-incubated groups. The opposite effect was observed for unsaturated fatty acids (−24%, −23%, 2 h PQQ/PA vs. PA/PQQ 2 h, 24 h PQQ/PA vs. PA/PQQ 24 h, respectively, *p* < 0.05; Table A1).

#### 2.1.2. DAG Content and Composition in L6 Myotubes

Palmitate significantly elevated DAG content in muscle cells as compared to the control, specifically through the augmented level of saturated fatty acids (+46%, +51%, PA vs. control, *p* < 0.05; Figure 1b, Table A2).

Prolonged post-treatment with PQQ substantially elevated DAG content (+23%, PA/PQQ 24 h vs. PA, *p* < 0.05) as a result of an increase in both saturated and unsaturated fatty acids amount (+21%, +62%, PA/PQQ 24 h vs. PA, respectively, *p* < 0.05; Figure 1b, Table A2). The total level of DAG as well as saturated and unsaturated species was also significantly higher due to prolonged post-stimulation of L6 cells with PQQ as compared to short-term incubation (+41%, +42%, +47%, 24 h vs. 2 h of PA/PQQ, respectively, *p* < 0.05; Figure 1b, Table A2).

Short-term pre-treatment with PQQ did not significantly modify the level of DAG in L6 myotubes as compared to palmitate group. On the other hand, long-term pre-incubation considerably elevated the concentration of DAG in L6 myotubes as compared to palmitate group (+35%, 24 h PQQ/PA vs. PA, *p* < 0.05) following an increase in saturated fatty acids content (+37%, 24 h PQQ/PA vs. PA, *p* < 0.05; Figure 1b, Table A2). Furthermore, time-dependent differences in the effects of pre-treatment with PQQ were noticed in DAG (+23%, 24 h vs. 2 h PQQ/PA, *p* < 0.05) and the amount of saturated fatty acids (+28%, 24 h vs. 2 h PQQ/PA, *p* < 0.05), particularly regarding palmitic acid level (+34%, 24 h vs. 2 h PQQ/PA, *p* < 0.05; Figure 1b, Table A2).

Additionally, cells pre-treated with PQQ for 2 h had significantly higher DAG content than those post-incubated with PQQ (+26%, 2 h PQQ/PA vs. PA/PQQ 2 h, *p* < 0.05). Specifically, these changes concerned saturated fatty acids amount (+25%, 2 h PQQ/PA vs. PA/PQQ 2 h, *p* < 0.05). In the case of prolonged PQQ supplementation, the sequence of PQQ and palmitate treatment differentially influenced the content of unsaturated fatty acids that form DAG (−42%, 24 h PQQ/PA vs. PA/PQQ 24 h, *p* < 0.05; Figure 1b, Table A2).

#### 2.1.3. TAG Content and Composition in L6 Myotubes

L6 myotubes treated with palmitate exhibited substantially higher levels of TAG as compared to the control (+9-fold, PA vs. control, *p* < 0.05). Increased content of saturated fatty acids (+10-fold, PA vs. control, *p* < 0.05) resulted mainly from the elevated levels of myristic and palmitic acids (+97%, +17-fold, PA vs. control, respectively, *p* < 0.05). The parallel increase in unsaturated fatty acid content (+293%, PA vs. control, *p* < 0.05) was associated with augmented content of palmitoleic and linolenic acids (+10-fold, +91%, PA vs. control, respectively, *p* < 0.05; Figure 1c, Table A3).

Short-term post-incubation with PQQ reduced TAG level in muscle cells as compared to the palmitate group with a concurrent decrease in the level of saturated fatty acids (−20%, −23%, PA/PQQ 2 h vs. PA, respectively, *p* < 0.05). However, in this group the cellular level of unsaturated fatty acids was elevated, mainly because of increased palmitoleic acid content (+27%, +32%, PA/PQQ 2 h vs. PA, respectively, *p* < 0.05; Figure 1c, Table A3). Prolonged post-incubation with PQQ significantly increased intracellular TAG amount by raising the content of saturated and unsaturated fatty acids (+39%, +33%, +92%, PA/PQQ 24 h vs. PA, respectively, *p* < 0.05; Figure 1c, Table A3). Time-dependent differences were demonstrated for total TAG level together with saturated and unsaturated fatty acids amount (+70%, +72%, +51%, 24 h vs. 2 h of PA/PQQ, respectively, *p* < 0.05; Figure 1c, Table A3).

Neither short- nor long-term pre-palmitate PQQ supplementation changed TAG content in skeletal muscle cells as compared to the palmitate group. Nevertheless, the duration of PQQ incubation was relevant regarding unsaturated species level within the TAG pool. It was significantly lower after prolonged pre-palmitate PQQ exposure as compared to 2 h of cell stimulation, particularly due to the diminished amount of palmitoleic acid (−27%, −32%, 24 h vs. 2 h of PQQ/PA, respectively, *p* < 0.05; Figure 1c, Table A3).

Regarding the sequence-dependent differences, we noticed that 2 h pre-incubation of cells with PQQ was related to a substantially higher content of TAG and their saturated species (+21%, +23%, 2 h PQQ/PA vs. PA/PQQ 2 h, *p* < 0.05; Figure 1c, Table A3) as compared to the post-treatment model. On the other hand, L6 myotubes had lower TAG content under the conditions of prolonged pre-treatment with PQQ as compared to post-incubation effects, specifically because of the diminished amount of saturated and unsaturated species (−25%, −22%, −57%, 24 h PQQ/PA vs. PA/PQQ 24 h, respectively, *p* < 0.05; Figure 1c, Table A3).

To sum up, short-term PQQ action leads to a decrease in FFA (post- and pre-incubation) and TAG (pre-incubation) amount, whereas increased content of FFA (post-incubation), DAG (post- and pre-incubation) and TAG (post-incubation) content was demonstrated after 24 h of PQQ treatment.

#### 2.1.4. Sphingolipids Content and Composition in L6 Myotubes

**Figure 2:**
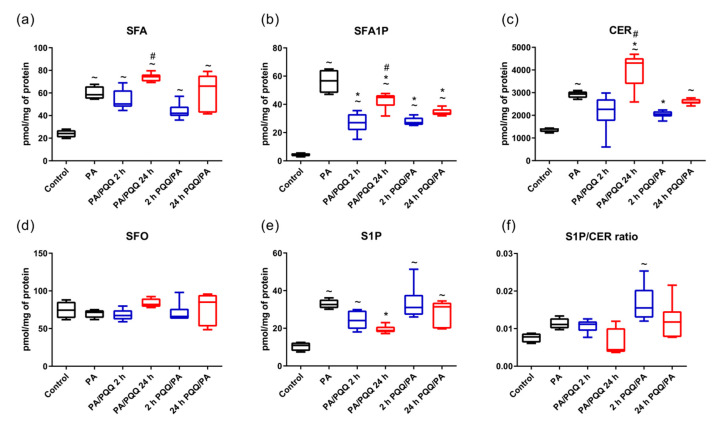
Effects of short- and long-time incubation with PQQ on the intracellular content of (**a**) sphinganine, (**b**) sphinganine-1-phosphate, (**c**) ceramide, (**d**) sphingosine, (**e**) sphingosine-1-phosphate, and (**f**) S1P/ceramides (CER) ratio. The results are based on six independent determinations. ANOVA with a post hoc Tukey’s test or the Kruskal–Wallis test followed by post hoc adjustment were applied. For the consistency of data presentation median (min–max) values were used. ^~^ *p* < 0.05, study groups versus control; * *p* < 0.05, PQQ-incubated groups versus palmitate group; ^#^ *p* < 0.05, time-dependent differences (long- vs. short-time incubation with PQQ). PA: palmitic acid; PQQ: pyrroloquinoline quinone.

Palmitate treatment significantly elevated CER level in muscle cells concomitantly with an increase in sphinganine (SFA) and sphinganine-1-phosphate (SFA1P) levels (+111%, +143%, +11-fold, PA vs. control, respectively, *p* < 0.05; Figure 2a–c). Moreover, increased amount of S1P was observed after palmitic acid exposure (+198%, PA vs. control, *p* < 0.05; Figure 2e), although S1P/CER ratio did not change considerably (Figure 2f).

Ceramide level was increased under the conditions of prolonged post-treatment with 0.5 µM of PQQ followed by a significant decrease in SFA1P and S1P concentration (+37%, −24%, −41%, PA/PQQ 24 h vs. PA, respectively, *p* < 0.05; Figure 2b,c,e). Furthermore, the cellular content of CER declined in response to 2 h pre-incubation with PQQ (−30%, 2 h PQQ/PA vs. PA, *p* < 0.05; Figure 2c). A reduced content of SFA1P was also noticed after short-time post-incubation with PQQ (−52%, PA/PQQ 24 h vs. PA, *p* < 0.05) and in both periods of PQQ pre-treatment (−51%, −39%, 2 h and 24 h of PQQ/PA vs. PA, respectively, *p* < 0.05; Figure 2b). Time-dependent differences in the effects of PQQ were demonstrated only in the post-incubation model for SFA, SFA1P, and CER cellular content (+48%, +66%, +90%, 24 h vs. 2 h of PA/PQQ, respectively, *p* < 0.05; Figure 2a–c). The level of sphingosine (SFO) in L6 myotubes did not change after PQQ incubation (Figure 2d).

### 2.2. The Impact of Pre- and Post-Incubation with PQQ on the Protein-Mediated Fatty Acid Transport

#### 2.2.1. The Impact of PQQ on the Expression of Fatty Acid Transporters at the Transcript (mRNA) and Protein Levels in L6 Myotubes

In order to understand the role of PQQ in cellular lipid metabolism, we studied both the magnitude of fatty acid uptake and the expression of various fatty acid transporting proteins that facilitate the entry of fatty acids to the cell.

**Figure 3:**
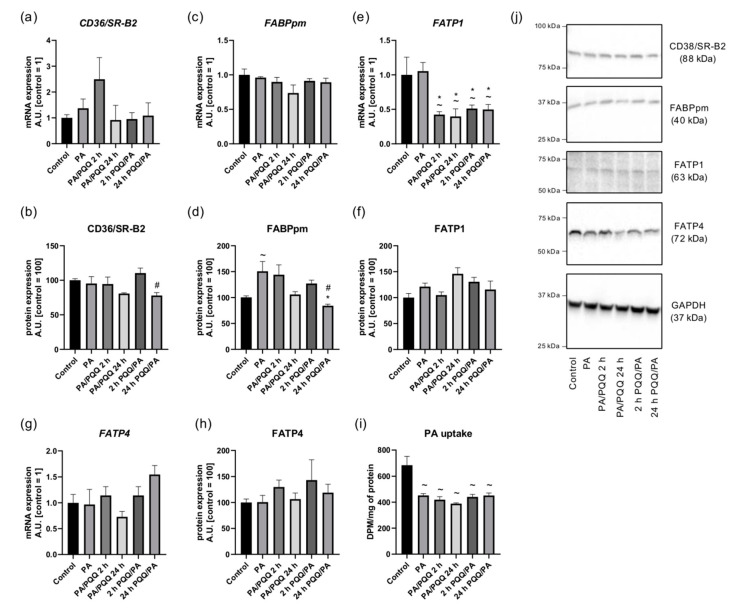
Effects of short- and long-time incubation with PQQ on the mRNA and protein levels of (**a**,**b**) cluster of differentiation 36/scavenger receptor class B protein, (**c**,**d**) plasma membrane-associated fatty acid binding protein, (**e**,**f**) fatty acid transport protein 1, (**g**,**h**) fatty acid transport protein 4 as well as (**i**) palmitic acid uptake. (**j**) Representative Western Blot data. Unless stated otherwise, the control is set as 1 (mRNA) or 100 (protein). The values are presented as mean ± SEM based on six independent determinations. ANOVA with a post hoc Tukey’s test was applied. ^~^ *p* < 0.05, study groups versus control; * *p* < 0.05, PQQ-incubated groups versus palmitate group; ^#^ *p* < 0.05, time-dependent differences (long- vs. short-time incubation with PQQ). ANOVA: analysis of variance; GAPDH: glyceraldehyde 3-phosphate dehydrogenase; mRNA: messenger RNA; PA: palmitic acid; PQQ: pyrroloquinoline quinone.

Palmitate treatment did not change mRNA level of any of the studied fatty acid transporting proteins (i.e., *CD36/SR-B2*, *FABPpm*, *FATP1*, *FATP4*), and solely a substantial increase in FABPpm protein content was noticed (+50%, PA vs. control, *p* < 0.05, Figure 3a–h,j).

With respect to PQQ exposure, we noticed a decrease of about 50–60% in the mRNA level of *FATP1* in all of the studied groups, while for the rest fatty acid transporters we did not observe any statistically significant differences. The protein content of all transporting proteins was similar in PQQ-exposed groups as compared to palmitate group, except for prolonged PQQ pre-incubation, where a noticeable drop in FABPpm expression was demonstrated (−44%, 24 h PQQ/PA vs. PA, *p* < 0.05; Figure 3a–h,j). Furthermore, we observed a time-dependent decrease in the protein expression of CD36/SR-B2 and FABPpm in the groups of pre-incubation with PQQ (CD36/SR-B2: −29%, FABPpm: −34%, 24 h vs. 2 h of PQQ/PA, *p* < 0.05; Figure 3b,d,j).

**Figure 4:**
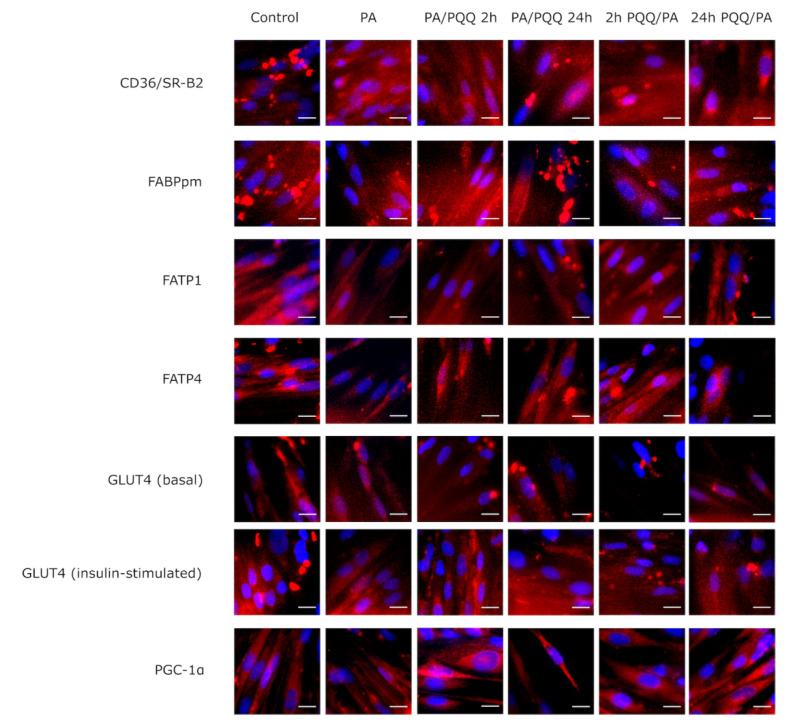
Effects of short- and long-time incubation with PQQ on the intracellular localization of fatty acid translocase/CD36 (CD36/SR-B2), plasma membrane-associated fatty acid binding protein (FABPpm), fatty acid transport protein 1 (FATP1), fatty acid transport protein 4 (FATP4), glucose transporter 4 (GLUT4) and peroxisome proliferator-activated γ co-activator 1α (PGC-1α). The fluorescence intensities of stained cells were analyzed using a BD Pathway 855 confocal microscope with a 40× (0.75 NA) objective. Scale bars (bottom right) are 15 μm. PA: palmitic acid; PQQ: pyrroloquinoline quinone.

As visualized in the immunofluorescence staining, fatty acid transporters were accumulated within intracellular depots or dispersed throughout the cell interior in L6 myotubes. Palmitate treatment could induce the redistribution of CD36/SR-B2 and FABPpm from cellular stores to cytoplasm and plasma membrane as also observed in PQQ-exposed groups (Figure 4). Our findings are in agreement with the studies supporting a primary role of these two proteins in controlling fatty acid transport in skeletal muscle cells [30], although PQQ seems not to exert any additional modifications.

#### 2.2.2. The Impact of PQQ on Palmitic Acid Uptake in L6 Myotubes

Due to the lack of observed differences in the protein expression of fatty acid transporters in the PQQ treated L6 myotubes it is hypothesized that fatty acid uptake did not change in these cells. Indeed, the intracellular uptake of radioactively labeled palmitic acid was not favored by PQQ, even though palmitate treatment decreased its uptake in all studied groups (−34%, −39%, −43%, −36%, −34%, PA, 2 h and 24 h of PA/PQQ, 2 h and 24 h of PQQ/PA vs. control, respectively, *p* < 0.05; Figure 3i).

### 2.3. The Impact of Pre- and Post-Incubation with PQQ on the Expression of Enzymes Involved in Substrate Metabolism

**Figure 5:**
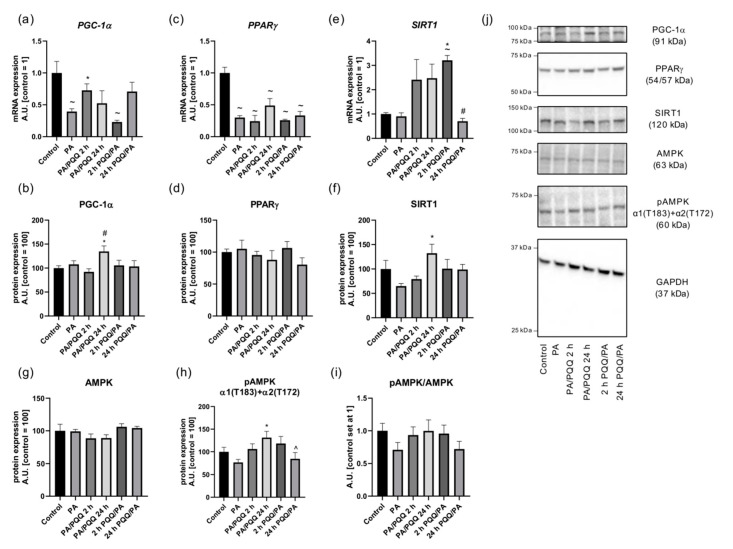
Effects of short- and long-time incubation with PQQ on the mRNA and protein levels of (**a**,**b**) peroxisome proliferator activated receptor γ co-activator 1α, (**c**,**d**) peroxisome proliferator activated receptor γ, (**e**,**f**) sirtuin 1, (**g**) 5′AMP-activated protein kinase, (**h**) phosphorylated 5′AMP-activated protein kinase and (**i**) pAMPK/AMPK ratio in L6 myotubes. (**j**) Representative Western Blot data. The control is set as 1 (mRNA, ratio) or 100 (protein). The values are presented as mean ± SEM and are based on six independent determinations. ANOVA with a post hoc Tukey’s test was applied. ^~^ *p* < 0.05, study groups versus control; * *p* < 0.05, PQQ-incubated groups versus palmitate group; ^#^ *p* < 0.05, time-dependent differences (long- vs. short-time incubation with PQQ); ^ *p* < 0.05, sequence-dependent differences (pre-incubation with PQQ vs. post-incubation with PQQ). ANOVA: analysis of variance; GAPDH: glyceraldehyde 3-phosphate dehydrogenase; mRNA: messenger RNA; PA: palmitic acid; PQQ: pyrroloquinoline quinone.

In order to evaluate whether PQQ treatment influenced the levels of metabolic enzymes, we examined the effect of palmitate and PQQ on the expression of various oxidative proteins reflecting the mitochondrial function. A statistically significant decrease in *PGC-1α* transcript level was demonstrated for palmitate group as well as cells pre-incubated with PQQ for 2 h (−61%, −77%, 2 h PQQ/PA vs. control, respectively, *p* < 0.05). Exposure of L6 myotubes to PQQ significantly elevated *PGC-1α* expression at mRNA level in the case of 2 h post-incubation with PQQ (+35%, PA/PQQ 2 h vs. PA, *p* < 0.05; Figure 5a), while its protein content increased in response to long-term post-treatment (+46%, PA/PQQ 24 h vs. PA, *p* < 0.05). Therefore, we assume that 2 h were insufficient to affect PGC-1α protein level. Moreover, time-dependent difference in the protein level of PGC-1α was observed for post-incubation with PQQ (+65%, 24 h vs. 2 h of PA/PQQ, *p* < 0.05; Figure 5b,j). As illustrated on Figure 4, subcellular localization of PGC-1α was mostly cytoplasmic in the control and palmitate groups, while PQQ induced its subcellular distribution to the cell nucleus.

We noticed that palmitate-treated groups had significantly lower level of *PPARγ* transcript as compared to the control cells (−70%, −76%, −51%, −77%, −67%; PA, 2 h and 24 h of PA/PQQ, 2 h and 24 h of PQQ/PA vs. control, respectively, *p* < 0.05; Figure 5c). However, there were no PQQ-dependent changes in the mRNA and protein expression of PPARγ as compared to the palmitate group (Figure 5c,d,j).

Both SIRT1 and AMPK play a major role in regulating fatty acid oxidation, but it remains unknown whether the treatment of muscle cells with PQQ alters the expression of these enzymes. In general, AMPK activates catabolic pathways, thus reducing intracellular AMP/ATP ratio and further stimulating SIRT1 activity through elevated cellular NAD^+^ level [31]. At the same time, it has been suggested that SIRT1 promotes the phosphorylation of AMPK α catalytic subunit (Thr172) through the action of liver kinase B1 [32]. In our study, *SIRT1* transcript content markedly increased after short-term of PQQ pre-incubation (+254%, 2 h PQQ/PA vs. PA, respectively, *p* < 0.05). The mRNA expression of *SIRT1* was significantly lower after prolonged pre-incubation with PQQ as compared to the short-term pre-treatment conditions (−78%, 24 h vs. 2 h of PQQ/PA, *p* < 0.05; Figure 5e). SIRT1 protein level was elevated as a consequence of 24 h of PQQ post-incubation (+104%, PA/PQQ 24 h vs. PA, *p* < 0.05; Figure 5f,j). There were no alterations in AMPK protein level in response to palmitic acid or PQQ incubation (Figure 5g,j), although pAMPK α1(T183)+α2(T172) expression substantially increased after prolonged post-treatment with PQQ (+31%, PA/PQQ 24 h vs. PA, *p* < 0.05). Furthermore, there were significant differences between 24 h of pre- and post-incubation with PQQ (−35%, 24 h PQQ/PA vs. PA/PQQ 24 h, *p* < 0.05; Figure 5h,j). Nevertheless, no significant changes in pAMPK/AMPK ratio were observed after PQQ exposure (Figure 5i).

**Figure 6:**
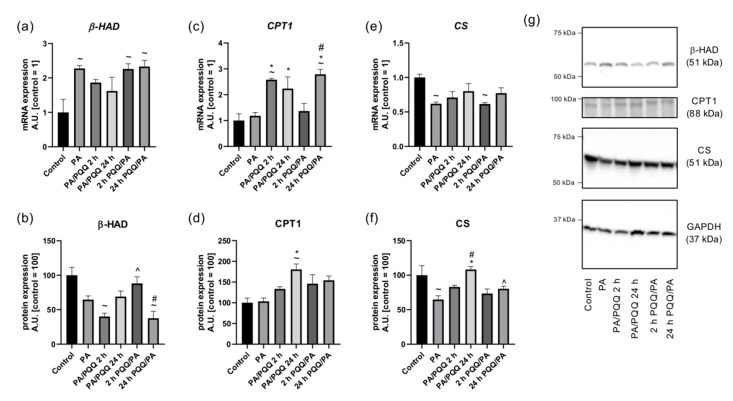
Effects of short- and long-time incubation with PQQ on the mRNA and protein levels of (**a**,**b**) 3-hydroxyacyl-CoA dehydrogenase, (**c**,**d**) carnitine palmitoyltransferase I, and (**e**,**f**) citrate synthase in L6 myotubes. (**g**) Representative Western Blot data. The control is set as 1 (mRNA) or 100 (protein). The values are presented as mean ± SEM and are based on six independent determinations. ANOVA with a post hoc Tukey’s test was applied. ^~^ *p* < 0.05, study groups versus control; * *p* < 0.05, PQQ-incubated groups versus palmitate group; ^#^ *p* < 0.05, time-dependent differences (long- vs. short-time incubation with PQQ); ^ *p* < 0.05, sequence-dependent differences (pre-incubation with PQQ vs. post-incubation with PQQ). ANOVA: analysis of variance; GAPDH: glyceraldehyde 3-phosphate, mRNA: messenger RNA; PA: palmitic acid; PQQ: pyrroloquinoline quinone.

Next, we determined the expression of enzymes which could serve as downstream effectors of PGC-1α, SIRT1, PPARγ and AMPK, being responsible for mitochondrial delivery and utilization of fatty acids. *β-HAD* transcript levels were stable in groups co-cultured with palmitate and PQQ, although some time- and sequence-dependent differences in their protein content were observed (Figure 6a,b,g). The discrepancy between the results obtained at the transcript and protein levels after PA exposure could be explained by a delay in protein synthesis or a short half-life for the protein (~2 h) [33].

CPT1 expression was elevated in PQQ-treated groups reaching statistical significance for cells post-incubated with 0.5 µM of PQQ for 24 h at both mRNA and protein levels (+89%, +75%, PA/PQQ 24 h vs. PA, *p* < 0.05; Figure 6c,d,g). An increase in *CPT1* transcript amount was also noticed after 2 h of PQQ post-treatment (+118%, PA/PQQ 2 h vs. PA, *p* < 0.05) and 24 h of PQQ pre-incubation (+136%, 24 h PQQ/PA vs. PA, *p* < 0.05). Furthermore, prolonged pre-treatment with PQQ resulted in a substantially higher level of *CPT1* mRNA than short-term pre-incubation (+104%, 24 h vs. 2 h of PQQ/PA, *p* < 0.05; Figure 6c).

The transcript content of *CS* remained constant after PQQ treatment, although an increase in its protein amount was demonstrated in the case of prolonged post-incubation with PQQ (+67%, PA/PQQ 24 h vs. PA, *p* < 0.05). Additionally, there were time- and sequence of administration-dependent differences in the protein expression of CS (Figure 6e,f,g).

**Figure 7:**
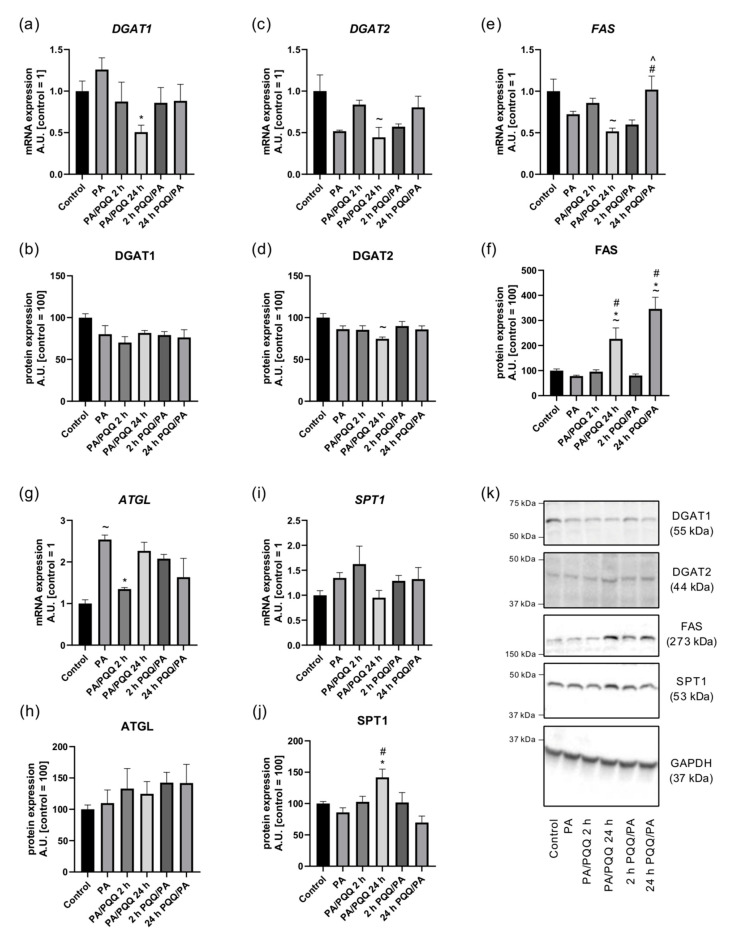
Effects of short- and long-time incubation with PQQ on the mRNA and protein levels of (**a**,**b**) diacylglycerol O-acyltransferase 1, (**c**,**d**) diacylglycerol O-acyltransferase 2, (**e**,**f**) fatty acid synthase, (**g**,**h**) adipose triglyceride lipase, and (**i**,**j**) serine palmitoyltransferase, long chain base subunit 1 in L6 myotubes. (**k**) Representative Western Blot data. The control is set as 1 (mRNA) or 100 (protein). The values are presented as mean ± SEM and are based on six independent determinations. ANOVA with a post hoc Tukey’s test was applied. ^~^ *p* < 0.05, study groups versus control; * *p* < 0.05, PQQ-incubated groups versus palmitate group; ^#^ *p* < 0.05, time-dependent differences (long- vs. short-time incubation with PQQ); ^ *p* < 0.05, sequence-dependent differences (pre-incubation with PQQ vs. post-incubation with PQQ). ANOVA: analysis of variance; GAPDH: glyceraldehyde 3-phosphate dehydrogenase; mRNA: messenger RNA; PA: palmitic acid; PQQ: pyrroloquinoline quinone.

In the next step, we focused on the enzymes engaged in lipogenesis. DGAT1 is dominant in skeletal muscle and generates TAG from fatty acids that are exogenously supplied, de novo synthesized or derived from lipolysis, whereas DGAT2 uses specifically glycerol-3-phosphate-derived fatty acids as a substrate [34]. A decrease in *DGAT1* mRNA content was observed after prolonged PQQ post-incubation (−49%, PA/PQQ 24 h vs. PA, *p* < 0.05; Figure 7a), while PQQ exposure did not modulate *DGAT2* mRNA expression (Figure 7c). We did not notice any alterations in the protein expression of DGAT1 and DGAT2 due to PQQ action as compared to the palmitate group (Figure 7b,d,k).

To gain further insight into molecular mechanisms behind PQQ-stimulated lipid profile we assessed FAS level in L6 myotubes. An increase in *FAS* transcript content was noticed for 24 h of PQQ pre-incubation as compared to short-term PQQ pre-treatment (+70%, 24 h vs. 2 h of PQQ/PA, *p* < 0.05) and to 24 h post-incubation (+97%, 24 h PQQ/PA vs. PA/PQQ 24 h, *p* < 0.05). A correspondingly marked increase in the protein expression of FAS was observed in the case of prolonged pre-treatment with PQQ (+342%, 24 h PQQ/PA vs. PA, *p* < 0.05). In the group post-treated with PQQ for 24 h the level of FAS protein was also elevated (+189%, PA/PQQ 24 h vs. PA, *p* < 0.05). In the models of post- and pre-palmitate PQQ exposure, time-dependent differences in FAS protein expression were demonstrated (+237%, 24 h vs. 2 h of PA/PQQ; +431%, 24 h vs. 2 h of PQQ/PA, *p* < 0.05; Figure 7e,f,k).

With regards to the principal enzyme responsible for TAG lipolysis, the increased mRNA level of *ATGL* resulting from palmitic acid exposure (+154%, PA vs. control, *p* < 0.05) was reversed solely by 2 h of post-incubation with PQQ (−47%, PA/PQQ 2 h vs. PA, *p* < 0.05; Figure 7g). The protein expression of ATGL, however, did not alter significantly in the presence of PQQ (Figure 7h,k).

Eventually, we investigated the effects of PQQ on the content of SPT1, the main enzyme engaged in de novo CER synthesis. *SPT1* gene expression did not change considerably (Figure 7i), while SPT1 protein content was elevated as a consequence of prolonged post-treatment with PQQ (+65%, PA/PQQ 24 h vs. PA, *p* < 0.05; Figure 7j,k).

Importantly, the regulation of the transcription and translation processes, multifactorial, and separated within subcellular compartments, can lead to the dissonance between mRNA and protein expressions. The level of mRNA depends on its stability and transcriptional rate, whereas protein content is influenced by translation rates determined by mRNA sequence, ribosome availability, binding of proteins to regulatory elements, as well as protein-specific delay in synthesis and the ‘translation on demand’ mechanism [35]. Moreover, it should be borne in mind that mRNAs and proteins are regulated with separated temporal patterns [36]. Nevertheless, it is evident that most PQQ-dependent alterations in the expression of metabolic enzymes are linked to PA/PQQ 24 h group, including elevated protein amount of PGC-1α, SIRT1, pAMPK, CPT1, CS, FAS, and SPT1.

### 2.4. The Impact of PQQ Stimulation on Insulin Sensitivity

**Figure 8:**
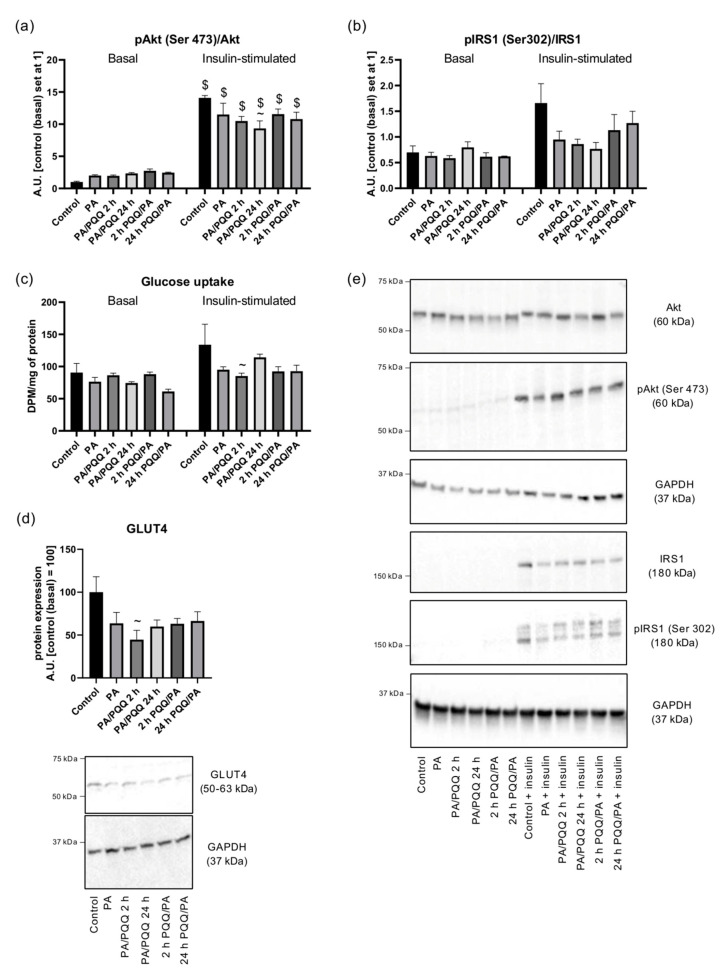
Effects of short- and long-time incubation with PQQ on the protein expression of (**a**) pAkt/Akt ratio, (**b**) pIRS1/ insulin receptor substrate (IRS1) ratio, (**c**) glucose uptake, and (**d**) glucose transporter 4 expression in L6 myotubes. (**e**) Representative Western Blot data. Unless stated otherwise, the control is set as 100 (protein content) or 1 (ratio). The values are presented as mean  ±  SEM and are based on six independent determinations. ANOVA with a post hoc Tukey’s test was applied. ^~^ *p* < 0.05, study groups versus control; $ *p* < 0.05, insulin-stimulated versus basal conditions. ANOVA: analysis of variance; GAPDH: glyceraldehyde 3-phosphate dehydrogenase; PA: palmitic acid; PQQ: pyrroloquinoline quinone.

**Figure A1:**
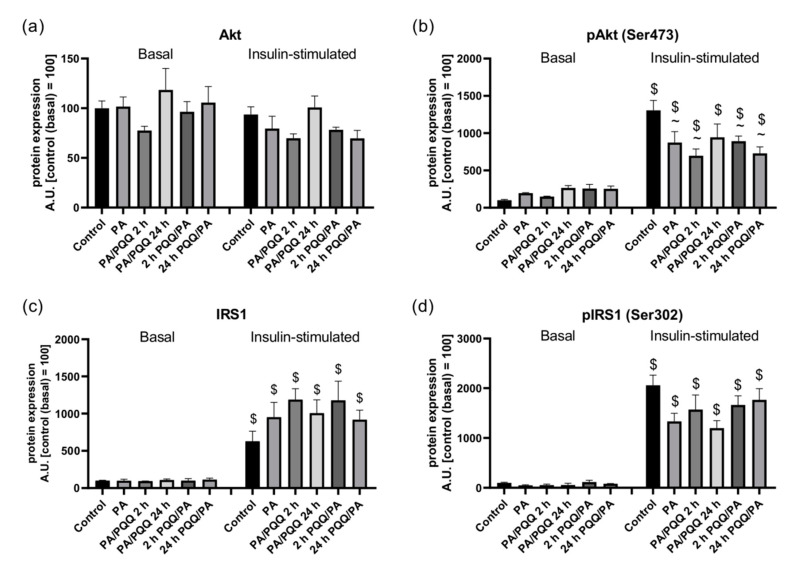
Effects of short- and long-time incubation with PQQ on the protein expression of (**a**) Akt, (**b**) pAkt (Ser 473), (**c**) IRS1, (**d**) pIRS1 (Ser302) in L6 myotubes. Control is set as 100 and the values are presented as mean  ±  SEM based on six independent determinations. ANOVA with a post hoc Tukey’s test was applied. ^~^ *p* < 0.05, study groups versus control; $ *p* < 0.05, insulin-stimulated versus basal conditions. ANOVA: analysis of variance; GAPDH: glyceraldehyde 3-phosphate dehydrogenase; PA: palmitic acid; PQQ: pyrroloquinoline quinone.

Next, we examined the effects of 16 h palmitic acid exposure on insulin sensitivity in L6 myotubes. Palmitate treatment did not alter Akt and IRS1 protein expressions as compared to control cells (Figure A1a,c), although we observed a diminished insulin-dependent Akt (−33%, PA vs. control, *p* < 0.05; Figure A1b) and IRS1 phosphorylation (−35%, PA vs. control, *p* > 0.05; Figure A1d) with a simultaneous slight decrease in pAkt/Akt and pIRS1/IRS1 ratios, and a tendency towards reduced insulin-stimulated glucose uptake (−18%, −29%, −43%, PA vs. control, respectively, *p* > 0.05; Figure 8a–c,e) in the palmitate group. Similarly to our observations, palmitate treatment (500 µM; 22 h) impaired insulin-stimulated phosphorylation of Akt at both Thr308 and Ser473 (about 25% decrease) in another study performed on L6 myotubes [37]. Additionally, a reduced GLUT4 expression was noticed due to palmitate administration (−36%, PA vs. control, *p* > 0.05; Figure 8d) further visualized by immunofluorescence bioimaging (Figure 4). Other studies showed palmitate-dependent decline in insulin-stimulated GLUT4 translocation to the plasma membrane in experiments with the use of the L6 myotube model with stable transfection of myc-tagged GLUT4 (L6-GLUT4myc) [37]. However, we did not confirm this by immunostaining (Figure 4), presumably because of low initial expression of GLUT4 and/or a small range of changes triggered by palmitic acid.

Finally, we analyzed the effects of PQQ supplementation in terms of insulin responsiveness. The expression of Akt remained comparable between the studied groups (Figure A1a), although pAkt (Ser473) level was elevated due to insulin action as compared to basal conditions (control: +12-fold; PA: +346%; PA/PQQ 2 h: +370%; PA/PQQ 24 h: +255%; 2 h PQQ/PA: +246%; 24 h PQQ/PA: +188%, insulin-stimulated vs. basal, *p* < 0.05; Figure A1b). Simultaneously, pAkt/Akt ratio was elevated after insulin-stimulation (control: +13-fold; PA: +475%; PA/PQQ 2 h: +735%; PA/PQQ 24 h: +298%; 2 h PQQ/PA: +321%; 24 h PQQ/PA: +340%, insulin-stimulated vs. basal, *p* < 0.05; Figure 8a,e). Insulin significantly elevated IRS1 (control: +529%; PA: +8.5-fold; PA/PQQ 2 h: +12-fold; PA/PQQ 24 h: +10-fold; 2 h PQQ/PA: +11-fold; 24 h PQQ/PA: +9-fold, insulin-stimulated vs. basal, *p* < 0.05; Figure 8e or Figure A1c) and pIRS1 (Ser 302; control: +19-fold; PA: +13-fold; PA/PQQ 2 h: +16-fold; PA/PQQ 24 h: +12-fold; 2 h PQQ/PA: +17-fold; 24 h PQQ/PA: +18-fold, insulin-stimulated vs. basal, *p* < 0.05; Figure 8e or Figure A1d) levels, however no statistical differences were observed as a result of PQQ action. The measurement of radioactively labeled glucose uptake in L6 cells also did not show any significant alterations in the response to PQQ exposure as compared to palmitate group, although, after insulin exposure, it was substantially lower than in control cells reaching statistical significance for short-term post-incubation with PQQ (−36%, PA/PQQ 2 h vs. control, *p* < 0.05; Figure 8c). In cells co-cultured with palmitic acid and PQQ the expression of GLUT4 was stable as compared to the palmitate group (Figure 8d). Moreover, we did not observe any changes in the cellular redistribution pattern of GLUT4 after PQQ supplementation in both basal and insulin-stimulated groups as compared to the control (Figure 4). Therefore, based on these outcomes we cannot support the hypothesis of insulin-sensitizing effects of PQQ.

## 3. Discussion

The dysfunctional regulation of lipid uptake and oxidation determines the pathogenesis of metabolic disorders, such as type 2 diabetes. Apart from exercise and nutrition, pharmacological interventions are applied to alleviate hyperglycemia and insulin resistance. PQQ is widely acknowledged as a potent anti-oxidative agent and essential regulator of energy metabolism in humans and rodents. To date, it is not clear whether PQQ is engaged in the regulation of lipid metabolism in the state of insulin resistance. Therefore, in the present study, we aimed to evaluate the potential role of PQQ as a regulator of lipid metabolism as well as an insulin-sensitizing agent in skeletal muscle cells challenged with high lipid provision that exceeded their energy demand. The study focused on three major constituents ensuring appropriate insulin tolerance: (1) mitochondrial function in regard to energy metabolism, (2) fatty acids transport and metabolism as well as (3) intracellular glucose uptake.

In the previous study, we demonstrated that PGC-1α expression in L6 myotubes is regulated in a dose- and time-dependent manner upon PQQ supplementation [29]. Much evidence also support a role of PQQ in mitochondrial biogenesis through the stimulation of transcriptional co-activator PGC-1α. The above was noticed in Hepa 1–6 cells [25], hepatic tissue of rats [38,39], SH-SY5Y neurons [40], denervated murine gastrocnemius muscles [41], and human muscles [42]. However, the results obtained at mRNA and protein levels for PGC-1α do not exactly correlate with each other [29,43]. It is well known that in some circumstances there is a poor relationship between the transcriptional (mRNA) and post-transcriptional (protein expression) measures. In general, only about 40% variability in tissue protein level is consistent with its mRNA level [44]. Herein, insulin-resistant L6 myotubes exhibited significantly elevated mRNA level after short-term post-incubation with PQQ, while its protein content increased after long-term post-stimulation of cells with PQQ. In the case of PGC-1α, a relatively short half-life for this protein, accounting for around 20 min, might be another reason for the disparity between the detected co-activator’s transcript and protein amount [45]. In all the studied groups, we cannot, however, exclude that PQQ stimulates posttranslational modifications regulating PGC-1α expression and function. For instance, PGC-1α may undergo phosphorylation by AMPK [46], while PQQ induced pAMPK and acetyl-CoA carboxylase (ACC; i.e., a signal of AMPK induction) expression in NIH/3T3 fibroblasts in a dose- and time-dependent manner [47]. In some studies, however, no evident connection between PGC-1α expression and AMPK or ACC phosphorylation status was shown in human skeletal muscles [48,49]. Additionally, the SIRT1-mediated PGC-1α deacetylation created its more stable and active form as well as modulated PGC-1α ability to interact with transcription factors [50]. In our study, we observed increased protein content of pAMPK and SIRT1 during the long-term post-incubation with PQQ. Accordingly, PGC-1α protein content was elevated in this group, which may contribute to the regulation of fatty acid utilization. Likewise, in basal conditions PQQ-dependent activation of SIRT1 was observed in HepG2 cells together with the elevated mRNA level of PGC-1α and other respiratory markers [43]. On the other hand, it was shown that PQQ concentrations of 10–100 nM rather stimulate SIRT1-dependent PGC-1α deacetylation and facilitate its nuclear translocation than affect its protein expression in NIH/3T3 fibroblasts [47]. Consistently, we observed such translocation in groups post-exposed to PQQ (2 h and 24 h), and pre-treated for 24 h. As stated above, the presented findings may support the hypothesis of pAMPK, SIRT1, and PGC-1α as major effectors for PQQ action in palmitate-treated muscle cells.

Mitochondria are fundamental metabolic organelles in the cell so their functional disturbances have been linked to insulin resistance. Recent studies have found that PQQ might be the regulator of mitochondrial function, as evidenced by reduced respiratory control ratios and respiratory quotients in PQQ-deprived mice [51] that are in agreement with diminished mtDNA/nuclear DNA ratios as compared to rats fed a basal PQQ(+) diet [52]. It is noteworthy that the reduction in carnitine palmitoyltransferase (CPT) level observed in diabetes is a rate-limiting step underlying the delivery of long-chain fatty acids to mitochondria [53], while PGC-1α [54] and SIRT1 [55] may regulate its expression. Indeed, the overexpression of PGC-1α upregulates long-chain and very-long-chain fatty acid oxidation through coordinated increase in peroxisomal and mitochondrial functions [25,39,41,56]. Bauerly et al. had also demonstrated that, in basal state, a diet enriched in 6 nmol/g of PQQ was sufficient to intensify PPARα, FABP, and acyl CoA oxidase (AOX) expression in heart and liver tissue [52]. Importantly, the authors confirmed higher PQQ amounts in plasma and tissue (i.e., liver, heart) of animals fed PQQ-rich diets [52]. Once stimulated with PQQ, cells develop features of enhanced mitochondrial oxidative potential [25,52], thus we examined whether it also occurs in cells challenged with high dose of palmitic acid. Our data indicate considerable alterations in the expression of several enzymes promoting glucose and fatty acid oxidation under prolonged PQQ post-treatment (i.e., increased expression of PGC-1α, SIRT1, pAMPK, CPT1, and CS), which could eventually enhance ATP production through tricarboxylic acid (TCA) cycle and oxidative phosphorylation [57]. Therefore, it seems that the impact of PQQ on mitochondrial function varies in time- and a sequence-dependent manner (pre- or post-incubation). It is also possible that the effects accompanying PQQ administration may be time-limited, considering the gene expression patterns and transcriptional networks in rats fed PQQ repleted (PQQ−/+) and depleted (PQQ+/−) diets. The switch between PQQ addition/deprivation rapidly reversed or normalized the expression of the affected genes [39]. Additionally, the authors monitored PQQ levels and demonstrated a paralleled decrease in plasma PQQ amount after its short-term (48 h) depletion [39].

While intramyocellular lipids are crucial for energy metabolism and cell signaling, the obesity-related fatty acids overflow into non-oxidative pathways entails the heightening of the content of detrimental lipid intermediates. Previous studies have implicated abnormal deposition of CER and DAG in muscle cells as drivers of insulin resistance, although this relationship is not as straightforward as previously thought [58,59,60]. In this regard, strategies to counteract insulin resistance should elicit possibility to link muscular lipid utilization and insulin signaling improvement. Interestingly, Hoeks et al. also revealed that the overexpression of PQQ effector protein, PGC-1α, preferentially mobilizes mitochondrial oxidation of lipids instead of the use of carbohydrates as an energy substrate [61]. In the presence of palmitic acid, the impact of PQQ on the content of the selected lipid fractions appears to be time-dependent. Specifically, prolonged (24 h) PQQ post-treatment together with palmitate exposure exerted additive effect on TAG, DAG, and CER storage, while short-term (2 h) exposure resulted in reduced TAG and DAG amount and relatively unaltered CER content as compared to the palmitate group. Less pronounced changes were observed for PQQ pre-incubation, although the time-dependent differences were still noticeable (reduced FFA and CER in response to short-term exposure; elevated FFA and DAG due to 24 h PQQ supplementation). Despite profound alterations in lipid content, the expression of fatty acid transporters was stable in the group of post-incubation with PQQ. The reasons for such discrepancies between fatty acid transport and lipid content could involve the acceleration of passive diffusion of fatty acids (flip flop mechanism) due to their high concentration gradient across the plasma membrane [62,63]. Additionally, the translocation of fatty acid transporters from intracellular depots to the plasma membrane is often sufficient to couple increased extracellular concentration of fatty acids with their uptake without changes in the total pool of these proteins [64].

We observed no alterations in the level of proteins engaged in the final step of TAG synthesis (i.e., DGAT enzymes) after palmitate and PQQ interventions, despite the fact that TAG levels increased. It is probable that DGAT1 activity adjusted to the presence of high acyl-CoA levels [65], because of allosteric interaction of these compounds with N-terminal domain of DGAT1 [66]. PGC-1α could strengthen this effect since the study on the gain of function revealed that the co-activator stimulates also glucose-derived acetyl-CoA synthesis through the action of mitochondrial citrate carrier (SLC25A1) and ATP citrate lyase (ACLY) [67]. It is likely that the highest PGC-1α overexpression entailed the highest increase in TAG content in the PA/PQQ 24 h group. TAG lipolysis is catalyzed primarily by ATGL, whilst a slight increase in its protein expression in PQQ-exposed groups did not achieve statistical significance. Nevertheless, the elevated level of both DGAT1 and ATGL accompanied the 15-fold overexpression of PGC-1α caused by an adenoviral transfection in human primary myotubes [56]. We cannot ignore the possibility that PQQ may stimulate other routes of lipids production encompassing de novo synthesis, ceramide conversion to sphingomyelin or phospholipid breakdown. Importantly, cells undergoing prolonged stimulation with PQQ had significantly increased FAS protein content, suggesting a role of de novo lipogenesis in the accumulation of complex lipid fractions. Metabolic regulatory mechanisms observed for prolonged PQQ stimulation are similar to that of muscle-specific PGC-1α overexpression in animals fed high fat diet. For instance, increased activities of acetyl-CoA carboxylase (ACC) [67] and FAS [68,69] as well as elevated DAG content were present in that model, although, contrary to our findings, a comparable TAG and CER amounts were noticed in both of these studies [68,69]. The discrepant outcomes from animals and cellular models might be attributed to the activation of compensatory in vivo mechanisms to match fuel stores with demands, which are difficult to observe in a cell model. Furthermore, prolonged incubation with PQQ resulted in the increased CER generation as a consequence of elevated SPT1 level and the mobilization of SFA1P, which indicated the activation of de novo CER synthesis pathway. This is in line with elevated SPT activity following PQQ supplementation in hepatocytes [52]. Moreover, PQQ may modulate the salvage pathway since S1P content reduction was observed, although S1P/CER ratio did not change significantly.

Skeletal muscle lipid composition is thought to play a role in the development of insulin resistance since abundant content of saturated fatty acids correlates with lower insulin sensitivity [68,70,71]. It has been determined that unsaturated and shorter-chain fatty acids are preferentially mobilized as fuel source [72], although herein we observed either increased or unchanged degree of unsaturated fatty acids within FFA, DAG, and TAG pools. The highlighted effects suggest that PQQ predominantly induces the direction of saturated, instead of unsaturated, acyl species for further metabolic pathways when cells are exposed to a palmitic acid-rich environment. It is especially important since recent studies have emphasized the potential role of accumulated saturated TAG species as a primary markers of an early-stage insulin resistance instead of increased IMCL amount itself [73]. Furthermore, cells that had undergone prolonged PQQ treatment had higher levels of both saturated (pre- and post-incubation) and unsaturated DAG species (only post-incubation), thus resembling data found in endurance trained athletes [74].

Several reports demonstrated improvements in insulin tolerance in PQQ treated models. For instance, PQQ administration not only reduced glucose and increased insulin plasma concentration in streptozotocin (STZ)-treated animals that developed diabetes [75,76], but also positively modified the serum lipid profile by attenuating dyslipidemia, i.e., lowered levels of TAG, cholesterol, low-density lipoprotein cholesterol (LDL-C) and very-low-density lipoprotein cholesterol (VLDL-C). Moreover, elevated content of high-density lipoprotein (HDL-C) was observed in these animals [75]. Additionally, PQQ-mediated improvement in glucose tolerance was corroborated in type 2 diabetic KK-A^y^ mice through the oral glucose tolerance test (OGTT) test [77]. In line with this notion, experiments on the C2C12 cellular model revealed that 100 to 1000 nM of PQQ treatment stimulated insulin signaling through the inhibition of protein tyrosine phosphatase 1B (PTP1B) followed by increased phosphorylation of IRS1 (Tyr612), Akt (Ser473) and Erk1/2 (Thr202/Tyr204), despite their constant total protein expression. Consequently, GLUT4 translocation to the plasma membrane was observed although its total protein content has not changed [77]. In our study, however, pre- and post-incubation with PQQ did not modify the effect of palmitate exposure on glucose uptake and GLUT4 expression, except for prolonged PQQ post-incubation, in which pAkt/Akt ratio was decreased. Additionally, both total protein content of Akt and IRS, and their phosphorylated forms (Ser473 and Ser302, respectively) were not altered due to PQQ treatment in L6 muscle cells. These discrepancies could result from the experimental models used (basal state in C2C12 cells vs. insulin resistance in L6 myotubes) [77]. However, the theory that higher PQQ doses or increased incubation time would alleviate insulin resistance in cells overloaded with palmitate might be questioned. In biological fluids and amino acid-enriched solutions PQQ forms adducts with amino acids, exposed terminal amine (e.g., protein lysyl side chains) or alcohols, primarily imidazole pyrroloquinoline (IPQ) [78,79]. This process occurs both in vivo and in vitro [78,80,81], e.g., the content of authentic PQQ added to a diet is reduced by a half within 2 h when dissolved in sodium phosphate buffer at pH 7.0. Moreover, the acidic environment seems to accelerate this transformation [78]. In line with this, continuous PQQ administration could be crucial to enhance the level of its free form and exert the biological effects. Nevertheless, some studies contradict this hypothesis. For instance, despite the fact that an improved mitochondrial function was connected with reduced DAG and TAG plasma level in control rats fed PQQ-supplemented diet, PQQ doses that were 10–50 times higher than those found in the human diet and were used in that study failed to alter plasma glucose and insulin levels in both the control and type 2 diabetes animal models [52]. The fact that IPQ dissociates to PQQ with K_d_ = 10^−4^ [43] and the scarcity of data estimating equilibrium between PQQ and its derivatives make it difficult to develop the most effective strategy of PQQ treatment, and rather the cumulative effects of PQQ, IPQ, and other adducts could be assessed. Indeed, the currently available literature provides no data describing only PQQ action, i.e., after the elimination of its condensation products. Therefore, in most of PQQ-based studies it is hard to unambiguously separate a direct and indirect effects of the compound.

As mentioned above, the major limitation of this research includes lack of PQQ-derived products assessment. Few studies established that IPQ exhibits some PQQ properties (i.e., stimulation of DNA synthesis, antioxidative defense), although not all of them were attributed to this molecule (i.e., mitochondriogenesis) [43,80]. This might in turn alleviate the effects of PQQ, for instance decreasing steady-state NAD^+^ production, which seems to depend on the rate of lactate dehydrogenase (LDH)-PQQ complex formation. Eventually, conversion of pyruvate to lactate accelerates, what contributes to lower acetyl-CoA formation and diminished ATP production [57].

## 4. Materials and Methods

### 4.1. Cell Culture

The study was performed in differentiated rat-derived L6 skeletal muscle cells purchased from ATCC (American Type Culture Collection, Manassas, VA, USA). Myoblasts were incubated at 37 °C in humidified atmosphere containing 5% CO_2_ in high-glucose Dulbecco’s Modified Eagle Medium (DMEM, Pan Biotech, Aidenbach, Germany) supplemented with 10% Fetal Bovine Serum (FBS, Thermo Scientific, Waltham, MA, USA) and 1% antibiotic/antimycotic. At the confluency of approximately 60–80%, the cells were transferred to DMEM (4.5 g/L glucose) with 2% of horse serum in order to induce their differentiation into myotubes. After 10 days the cells were subjected to experiments (≥90% myoblasts were fused to form elongated myotubes as confirmed by a visual inspection using phase-contrast microscopy).

### 4.2. Cell Treatment

Prior the experiments, L6 myotubes were starved in serum-free DMEM (*w/o* glucose) for 3 h [82,83]. In order to evoke insulin resistance, L6 myotubes were exposed to high concentration of palmitic acid for 16 h. Briefly, palmitate stock was prepared by dissolving palmitate in a solution of absolute ethanol and 1 M NaOH, heating it to 70 °C and conjugating with 10% of bovine serum albumin (BSA, Sigma Aldrich, St. Louis, MO, USA). An aliquot of 8 mM palmitic acid solution was added to the culture medium to yield a final concentration of 0.5 mM.

Additionally, cells were co-cultured with a commercially available PQQ coenzyme (pyrroloquinoline quinone disodium salt, Sigma Aldrich, St. Louis, MO, USA). The dose and time of PQQ exposure were chosen based on our previous report [29] as well as literature analysis [25,51,77,84,85,86] thoroughly described in that paper. PQQ stock solution was prepared in water after heating at 37 °C and sonication, and stored at −20 °C. Immediately before the experiment, stock was dissolved in DMEM in order to obtain the PQQ concentration of 0.5 μM. In one of the groups, PQQ administration for 2 h or 24 h was performed before incubation with palmitate (0.5 mM) for the next 16 h (pre-treatment with PQQ; designation of groups: 2 h PQQ/PA, 24 h PQQ/PA). In the second set of experiments, cells were incubated with palmitate (0.5 mM, 16 h) and then treated with PQQ (post-treatment with PQQ; designation of groups: PA/PQQ 2 h, PA/PQQ 24 h).

### 4.3. RNA Isolation and Expression Analysis

The mRNA levels of selected genes were assessed by quantitative real-time PCR (qRT-PCR; Table A4). RNA was isolated from L6 myotubes using NucleoSpin RNA Plus (Aqua Lab, Warsaw, Poland) according to the manufacturer’s protocol. The quality of RNA was evaluated by measuring the absorbances at 260 and 280 nm wavelength. The synthesis of cDNA was performed using the EvoScript universal cDNA master kit (Roche Molecular Systems, Boston, MA, USA). The quantitative real time polymerase chain reaction (qRT-PCR) was carried out using the LightCycler 96 System with FastStart essential DNA green master (Roche Molecular Systems). PCR product specificity was verified by melting curve analysis carried out at the end of each reaction. The simultaneous determination of relative rat GAPDH gene expression was used as endogenous control. The mRNA levels of target genes were normalized to GAPDH and calculated according to Pfaffl method [87]. All samples were assayed in duplicate.

### 4.4. Western Blot Analysis

A routine Western blotting procedure [29] was applied to determine the expression of proteins of interest (i.e., PGC-1α, SIRT1, PPARγ, CD36/SR-B2, FABPpm, FATP1, FATP4, CPT1, β-HAD, CS, AMPK, pAMPK, ATGL, DGAT1, DGAT2, FAS, SPT1, GLUT4, Akt, pAkt, IRS, pIRS) in cell lysates. Briefly, cells were lysed in the radioimmunoprecipitation assay (RIPA; 50 mM Tris-HCl, 150 M NaCl, 1 mM EDTA, 1% NP-40, 0.25% Na-deoxycholate, 1 mM phenylmethylsulfonyl fluoride) buffer with protease and phosphatase inhibitors (Roche Diagnostics GmbH, Mannheim, Germany) and sonicated for 30 seconds at 4 °C. The total protein concentration was determined by means of a bicinchoninic acid (BCA) protein assay kit with bovine serum albumin (BSA) as a standard. Then, the samples were boiled at 95 °C for 10 min in a buffer containing 2-mercaptoethanol. The proteins (30 μg) were separated by 10% SDS-PAGE and wet transferred onto polyvinylidene fluoride (PVDF) membranes (0.2 μm pores, Bio-Rad, Hercules, CA, USA). Next, the membranes were blocked in Tris Buffered Saline + Tween 20 (TTBS buffer; 50 mM Tris-HCl, 130 mM NaCl, and 0.05% Tween-20) supplemented with 5% nonfat dry milk or 5% bovine serum albumin for 90 min at room temperature. After that, the membranes were immunoblotted overnight at 4 °C with the corresponding primary antibodies in a dilution of 1:500 or 1:1000. The primary antibodies were purchased from Santa Cruz Biotechnology (FATP1, sc-25541; SIRT1, sc-74465; PPARγ, sc-7196; AMPKα1, sc-398861; CPT1, sc-31128; DGAT2, sc-32400; ATGL, sc-365278; GAPDH, sc-25778), Novus Biologicals (PGC-1α, NBP1-04676; DGAT1, NB110-41487; GLUT4, NBP1-49533), Abcam (CD36/SR-B2, ab252922; FABPpm, ab153924; FATP4, ab200353; pAMPK α1(T183)+α2(T172), ab23875; β-HAD, ab230667; CS, ab129095; SPT1, ab176706) and Cell Signaling (Akt/PKB, #9272; pAkt/PKB (Ser473), #9271; IRS1, #2382; pIRS (Ser302), #2384; FAS, #3180). Subsequently, anti-rabbit, anti-mouse or anti-goat IgG horseradish peroxidase-conjugated secondary antibodies (1:3000; Santa Cruz Biotechnology, Dallas, TX, USA) were used to detect proteins. The protein bands were visualized using an enhanced chemiluminescence substrate (Thermo Scientific) and quantified by densitometry (Bio-Rad). The Ponceau S staining technique was used to confirm equal protein loading on the blot membrane. The protein expression (expressed in Optical Density Arbitrary Units) was normalized to GAPDH level. The control was set at 100 and the experimental groups were expressed relative to the control.

### 4.5. Immunofluorescence Staining and Bioimaging

The cells were seeded in BD Falcon™ 96-well black, clear-bottom tissue culture plates (BD Biosciences, San Jose, CA, USA) at 10,000 cells per well. After incubation, L6 cells were rinsed with PBS and fixed with a 3.7% formaldehyde solution (Sigma Aldrich) at room temperature for 10 min. Then, they were washed three times with PBS and permeabilized with 0.1% Triton X-100 at room temperature for 5 min. Afterwards, they were washed twice with PBS, and non-specific binding was blocked by incubation in 3% FBS at room temperature for 30 min. The cells were rinsed and incubated with anti-CD36/SR-B2, -FABPpm, -FATP1, -FATP4, -GLUT4, and -PGC-1α primary antibodies for night at room temperature. Next, they were washed three times with PBS and incubated with fluorescein isothiocyanate (FITC)-conjugated anti-rabbit secondary antibodies (1:500; BD Biosciences) for 60 min in the dark. After washing, nuclei were stained with Hoechst 33342 solution (2 μg/mL, blue) and analyzed using a BD Pathway 855 confocal microscope with a 40 × (0.75 NA) objective. The fluorescence intensities of the stained cells were evaluated, and images of FITC-labeled cells were acquired using a 488/10 excitation laser and a 515LP emission laser.

### 4.6. 9,10-[^3^H(N)]-Palmitic Acid Uptake

Palmitic acid uptake was evaluated according to the Chavez and Summers procedure [88]. Briefly, L6 myotubes were starved for 3 h before palmitic acid uptake assessment. Afterwards, myotubes were incubated for 20 min with either Krebs-Ringer buffer (KRB; 140 mM NaCl, 20 mM HEPES, 5 mM KCl, 2.5 mM MgSO_4_, 1 mM CaCl_2_) or KRB buffer supplemented with 100 nM insulin (Gensulin, Bioton, Poland). Subsequently, the cells were exposed to medium containing 2 mM palmitate complexed with 1% bovine serum albumin (Sigma Aldrich), with the addition of 9,10-[^3^H(N)]-palmitic acid (1 µCi/mL). After medium removal, the reaction was terminated with ice-cold PBS buffer and the cells were solubilized in 0.05 N NaOH. The resulting fluid was transferred to 5 mL scintillation vials and counted using a Packard TRI-CARB 1900 TR scintillation counter. Radioactivity was normalized concerning the protein concentration.

### 4.7. 2-[^3^H]-Deoxyglucose Uptake

The glucose uptake was conducted in L6 muscle cells after 3 h starvation (incubation in DMEM w/o glucose) for 3 h. Then, the myotubes were incubated for 20 min with either KRB buffer alone or supplemented with 100 nM insulin (Gensulin). The next step encompassed 10 min of incubation with 0.5 mM 2-deoxyglucose containing 1 µCi/mL of 2-[^3^H]-deoxyglucose. Eventually, the cells were rinsed 3 times with ice-cold PBS buffer and underwent solubilization in 0.05 N NaOH. The resulting fluid was transferred to 5 mL scintillation vials and taken for liquid scintillation counting. Radioactivity was normalized concerning the protein concentration.

### 4.8. Lipid Analysis

#### 4.8.1. Content and Fatty Acid Composition of Free Fatty Acids (FFA), Diacylglycerols (DAG) and Triacylglycerols (TAG)

Lipid fractions were extracted from L6 cells using a Bligh and Dyer method [89], i.e., the samples were transferred into glass tubes containing 2 mL of methanol with 0.01% butylated hydroxytoluene (antioxidant) and 4 mL of chloroform. Moreover, 100 μL of internal standard mixture (heptadecanoic acid, 1,2-diheptadecanoin and triheptadecanoin) (Sigma-Aldrich) was added. After 24 h, 1.5 mL of water was added to separate the lipid layer. Lipids dissolved in chloroform were evaporated under nitrogen stream (37 °C) and redissolved in 100 μL of chloroform-methanol solution (2/1, *v/v*). Thereafter, lipids were separated into specific fractions using thin-layer chromatography (TLC) (Kieselgel 60, 0.22 mm, Merck, Darmstadt, Germany) with a heptane:isopropyl ether:acetic acid (60:40:3, *v/v/v*) resolving solution. Dried silica plates were sprayed with 3′7′-dichlorofluorescin (0.2% solution in absolute methanol) and specific bands were visualized under ultraviolet light using standards on the plates. FFA were transmethylated with BF3/methanol [90], DAG fraction was eluted using chloroform-methanol solution (9/1, *v/v*) and the organic phase was redissolved in BF3/methanol solution. Finally, TAG fraction was eluted and methylated according to Christie [91]. Individual fatty acid methyl esters (FAMEs) in each fraction were identified and quantified according to the retention times of standards by gas liquid chromatography (Hewlett-Packard 5890 Series II gas chromatograph, HP-INNOWax capillary column; Agilent Technologies, Santa Clara, CA, USA). The total amounts of FFA, DAG, and TAG was estimated as the sum of the particular fatty acid species and expressed in nanomoles per milligram of protein.

#### 4.8.2. Content of Sphinganine (SFA), Sphinganine-1-phosphate (SFA1P), Sphingosine (SFO) and Sphingosine-1-phosphate (S1P)

The content of the selected sphingolipids was determined as described previously in detail [92]. Briefly, lipids were extracted from the samples in the presence of internal standards (10 pmol of C17-sphingosine and 30 pmol of C17-S1P; Avanti Polar Lipids, Alabaster, AL). The levels of S1P and SFA1P were evaluated indirectly after dephosphorylation to SFO and SFA, respectively, with the use of alkaline phosphatase (bovine intestinal mucosa, Sigma Aldrich). The chloroform fractions containing free sphingosine and sphinganine or dephosphorylated sphingoid bases were washed with alkalized water (pH adjusted to 10 with ammonium hydroxide) and then evaporated under a nitrogen stream. The dried lipid residues were redissolved in ethanol, converted to their o-phthalaldehyde derivatives and analyzed using a HPLC system (ProStar, Varian Inc., Palo Alto, CA, USA) equipped with a fluorescence detector and a C18 reversed-phase column (Varian Inc. OmniSpher 5, 4.6 × 150 mm). The isocratic eluent composition of acetonitrile (Merck): water (9:1, *v/v*) and a flow rate of 1 mL/min were used. The column temperature was maintained at 30 °C.

#### 4.8.3. Content of Ceramide (CER)

An aliquot of the chloroform phase containing lipids extracted as described above was transferred to a fresh tube containing 40 pmol of N-palmitoyl-d-erythro-sphingosine (C17 base) as an internal standard. The samples were then evaporated under a nitrogen stream, redissolved in 1.2 mL of 1 M KOH in 90% methanol and heated at 90 °C for 60 min to deacylate CER into SFO. The samples were partitioned by the addition of chloroform and water. The upper phase was discarded and the lower phase was evaporated under nitrogen and redissolved in ethanol. The content of free SFO released from CER was then analyzed using HPLC. The calibration curve was prepared using N-palmitoylsphingosine (Avanti Polar Lipids) as a standard. The chloroform extract used for the analysis of CER contained small amounts of free sphingoid bases. Therefore, the concentration of CER was corrected for the level of free SFO determined in the same sample.

### 4.9. Data Analysis and Statistics

Statistical analysis was performed using the GraphPad Prism 8 (GraphPad Software, La Jolla, CA, USA). The data were analyzed using analysis of variance (ANOVA) followed by a post hoc Tukey’s test for groups with normal distribution and homogeneity of variances. Whenever these assumptions did not hold, the nonparametric Kruskal–Wallis test with the following pairwise Wilcoxon test were applied. The threshold for statistical significance was *p* < 0.05.

## 5. Conclusions

**Figure 9:**
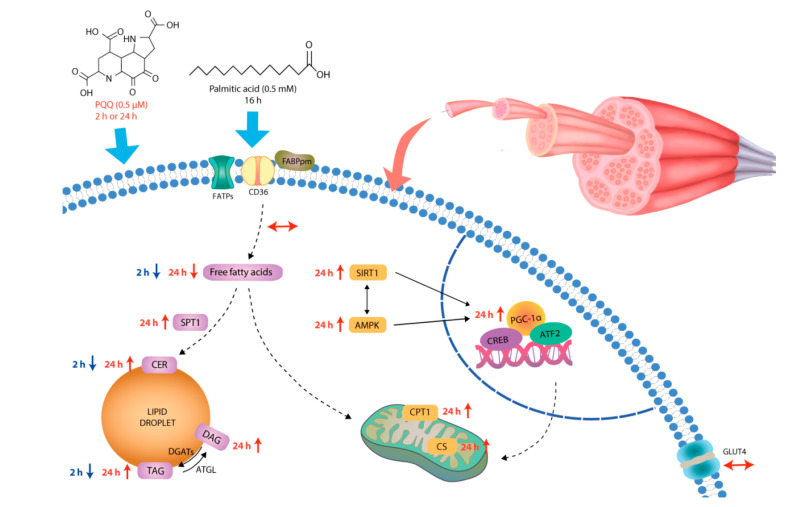
Effects of short- and long-time incubation with PQQ on energy substrate metabolism in L6 myotubes challenged with palmitate. ATF2: activating transcription factor 2; ATGL: adipose triglyceride lipase; AMPK: 5’AMP-activated protein kinase; CD36: cluster of differentiation 36; CER: ceramides; CPT1: carnitine palmitoyltransferase 1; CREB: cAMP-response element binding protein; CS: citrate synthase; DAG: diacylglycerols; DGATs: diacylglycerol O-acyltransferases; FABPpm: plasma membrane-associated fatty acid binding protein; FATPs: fatty acid transport proteins; GLUT4: glucose transporter type 4; PGC-1α: peroxisome proliferator-activated receptor γ co-activator 1α; PQQ: pyrroloquinoline quinone; SIRT1: sirtuin 1; SPT1: serine palmitoyltransferase, long chain base subunit 1.

A comparative approach between short- and long-term as well as pre- and post-incubation with PQQ was applied to evaluate lipid metabolism and insulin sensitivity in insulin-resistant L6 myotubes. It seems that the effects of short-term PQQ exposure are linked with catabolic processes as reflected by decreased FFA, DAG, SFA1P, or CER levels. More importantly, the sequence of prolonged PQQ administration (PA/PQQ vs. PQQ/PA) results in different metabolic outcomes. Prolonged post-treatment with PQQ (PA/PQQ 24 h) resulted in combined activation of fatty acid anabolism (i.e., accretion of DAG, TAG and CER pools) and oxidation (i.e., increased SIRT1, pAMPK, PGC-1α, CPT1, CS protein expression) to cope with augmented fuel availability. However, this adaptive response has not been observed in other groups (Figure 9). In conclusion, PQQ might concomitantly activate catabolic and anabolic processes in cells challenged with palmitate. The magnitude of these effects depends on the incubation time and the sequence of PQQ and palmitic acid administration.

## Abbreviations

AMPK	5’AMP-activated protein kinase
ATGL	adipose triglyceride lipase
β-HAD	3-hydroxyacyl-CoA dehydrogenase
CD36/SR-B2	cluster of differentiation 36/scavenger receptor class B protein
CER	ceramides
CPT1	carnitine palmitoyltransferase 1
CS	citrate synthase
DAG	diacylglycerols
DGAT1, 2	diacylglycerol O-acyltransferase 1, 2
FABPpm	plasma membrane-associated fatty acid binding protein
FAS	fatty acid synthase
FATP1, 4	fatty acid transport protein 1, 4
GAPDH	glyceraldehyde 3-phosphate dehydrogenase
GLUT4	glucose transporter type 4
IRS1	insulin receptor substrate 1
K_d_	dissociation constant
PGC-1α	peroxisome proliferator-activated receptor γ co-activator 1α
PPARγ	peroxisome proliferator activated receptor γ
PQQ	pyrroloquinoline quinone
S1P	sphingosine-1-phosphate
SFA	sphinganine
SFA1P	Sphinganine-1-phosphate
SFO	sphingosine
SIRT1	sirtuin 1
SPT1	serine palmitoyltransferase, long chain base subunit 1
TAG	triacylglycerols

## Appendix A

**Table A1:** Free fatty acids (FFA) content and composition in L6 muscle cells. The values (nmol mg^−1^ of protein) are expressed as: median (25–75%) based on six independent determinations. The Kruskal–Wallis test followed by a post hoc adjustment was applied. ^~^ *p* < 0.05, PA versus control; * *p* < 0.05, study group (co-cultured with PQQ) versus PA; ^#^ *p* < 0.05, time-dependent differences (long- versus short-time incubation with PQQ); ^ *p* < 0.05, sequence-dependent differences (pre-incubation with PQQ versus post-incubation with PQQ). PA: palmitic acid; PQQ: pyrroloquinoline quinone; SAT: saturated fatty acids; UNSAT: unsaturated fatty acids.

FFA	Control	PA	PA/PQQ 2 h	PA/PQQ 24 h	2 h PQQ/PA	24 h PQQ/PA
Myristic (14:0)	2.741 (2.457–2.922)	2.415 (2.277–2.574)	2.018 (1.800–2.164) ^~^	3.178 (3.037–3.369) *^,#^	2.292 (2.029–2.485)	2.994 (2.784–3.476) *
Palmitic (16:0)	6.409 (5.518–6.943)	32.578 (28.929–39.923) ^~^	8.746 (7.995–9.21) *	14.033 (12.671–17.434) ^~,*,#^	16.897 (16.013–20.602) ^~,^*^,^^	43.134 (40.166–49.454) ^~,^*^,#,^^
Palmitoleic (16:1)	0.366 (0.326–0.393)	1.075 (0.93–1.208) ^~^	1.328 (1.050–1.382) ^~^	1.444 (1.256–1.540) ^~,^*	0.801 (0.772–0.867) ^~,^*^,^^	0.95 (0.895–1.043) ^~,^^
Stearic (18:0)	3.325 (2.98–3.466)	1.884 (1.804–1.929) ^~^	1.769 (1.575–2.117) ^~^	4.293 (3.876–5.045) ^~,^*^,#^	2.983 (2.773–3.382) *^,^^	3.441 (3.256–3.604) *^,^^
Oleic (18:1n9c)	0.46 (0.408–0.496)	0.386 (0.344–0.43)	0.286 (0.235–0.307) ^~^	0.523 (0.454–0.598) ^#^	0.32 (0.28–0.38)	0.446 (0.385–0.513)
Linoleic (18:2n6c)	0.09 (0.082–0.103)	0.0935 (0.083–0.109)	0.068 (0.064–0.079)	0.102 (0.094–0.126) ^#^	0.089 (0.079–0.119)	0.086 (0.084–0.098)
Arachidic (20:0)	0.049 (0.045–0.053)	0.037 (0.034–0.045)	0.028 (0.024–0.035) ^~^	0.068 (0.066–0.071) ^~,^*^,#^	0.053 (0.043–0.056) ^	0.065 (0.061–0.073) ^~,^*^,#^
Linolenic (18n3)	0.047 (0.044–0.054)	0.033 (0.029–0.036) ^~^	0.034 (0.028–0.037) ^~^	0.061 (0.056–0.065) ^~,^*^,#^	0.04 (0.04–0.05) *^,^^	0.04 (0.036–0.043) ^
Behenic (22:0)	0.058 (0.053–0.07)	0.048 (0.044–0.049)	0.037 (0.028- 0.046) ^~^	0.067 (0.062–0.078) *^,#^	0.047 (0.043–0.05)	0.068 (0.059–0.082) *^,#^
Arachidonic (20:4n6)	0.032 (0.291–0.351)	0.262 (0.225–0.311)	0.287 (0.254–0.306)	0.3 (0.281–0.323)	0.209 (0.201–0.261) ^~^	0.31 (0.283–0.355) ^#^
Lignoceric (24:0)	0.047 (0.04–0.059)	0.048 (0.04–0.062)	0.022 (0.017–0.038)	0.057 (0.04–0.069)	0.041 (0.033–0.055)	0.07 (0.048–0.089)
Eicosapentaenoic (20:5n3)	0.116 (0.11–0.15)	0.124 (0.101–0.147)	0.118 (0.108–0.151)	0.126 (0.117–0.146)	0.121 (0.103–0.127)	0.129 (0.108–0.146)
Nervonic (24:1)	0.022 (0.02–0.025)	0.021 (0.019–0.022)	0.019 (0.016–0.021)	0.028 (0.024–0.034) ^~,^*	0.023 (0.02–0.024)	0.022 (0.019–0.024) ^
Docosahexaenoic (22:6n3)	0.039 (0.034–0.042)	0.029 (0.026–0.033)	0.028 (0.022–0.032) ^~^	0.052 (0.043–0.054) ^~,^*^,#^	0.024 (0.019–0.034) ^~^	0.035 (0.028–0.041) ^
SAT	12.549 (11.069–13.535)	36.911 (33.57–44.236) ^~^	12.541 (11.651–13.537) *	21.925 (19.958–25.2) ^~,^*^,#^	22.229 (21.053–26.613) ^~,^*^,^^	49.797 (46.406–57.004) ^~,^*^,#,^^
UNSAT	1.478 (1.365–1.583)	1.965 (1.849–2.262) ^~^	2.163 (1.778–2.303) ^~^	2.635 (2.373–2.821) ^~,^*^,#^	1.641 (1.580–1.818) ^	2.037 (1.878–2.158) ^~,^^
Total	14.027 (12.566–15.039)	38.876 (35.419–46.498) ^~^	14.616 (13.68–15.879) *	24.435 (22.519–28.019) ^~,^*^,#^	23.888 (22.652–28.358) ^~,^*^,^^	51.963 (48.428–58.986) ^~,^*^,#,^^

**Table A2:** Diacylglycerols (DAG) content and composition in L6 muscle cells. The values (nmol mg^−1^ of protein) are expressed as: median (25%–75%) based on six independent determinations. The Kruskal–Wallis test followed by a post hoc adjustment was applied. ^~^ *p* < 0.05, PA versus control; * *p* < 0.05, study group (co-cultured with PQQ) versus PA; ^#^ *p* < 0.05, time-dependent differences (long- versus short-time incubation with PQQ); ^ *p* < 0.05, sequence-dependent differences (pre-incubation with PQQ versus post-incubation with PQQ). PA: palmitic acid; PQQ: pyrroloquinoline quinone; SAT: saturated fatty acids; UNSAT: unsaturated fatty acids.

DAG	Control	PA	PA/PQQ 2 h	PA/PQQ 24 h	2 h PQQ/PA	24 h PQQ/PA
Myristic (14:0)	2.723 (1.603–3.664)	1.555 (1.443–2.324)	1.484 (1.399–1.705) ^~^	1.785 (1.639–2.199)	1.761 (1.508–2.039)	2.669 (2.245–2.890)
Palmitic (16:0)	10.485 (8.31–11.521)	23.453 (22.915–25.429) ^~^	19.308 (17.4993–22.116) ^~,^*	26.326 (25.46–27.384) ^~,#,^^	23.438 (22.863–24.559) ^~^	31.402 (30.049–31.638) ^~,^*^,#,^^
Palmitoleic (16:1)	0.415 (0.373–0.458)	0.767 (0.734–0.943) ^~^	1.065 (0.945–1.153) ^~,^*	1.439 (1.362–1.498) ^~,^*^,#,^^	0.764 (0.712–0.773) ^~,^^	0.687 (0.664–0.731) ^~,^^
Stearic (18:0)	5.362 (4.811–5.996)	4.055 (3.730–4.222)	3.824 (3.449–4.449) ^~^	6.566 (6.244–6.790) *^,#^	5.41 (5.077–5.724) *^,^^	5.224 (4.972–6.179) *
Oleic (18:1n9c)	0.895 (0.618–1.161)	0.469 (0.429–0.74)	0.449 (0.405–0.5225)	0.677 (0.644–0.725)	1.221 (0.941–1.376) *^,^^	0.755 (0.6473–1.201)
Linoleic (18:2n6c)	0.164 (0.145–0.225)	0.164 (0.117–0.213)	0.116 (0.103–0.14)	0.148 (0.146–0.173)	0.318 (0.245–0.375) ^	0.12 (0.104–0.219)
Arachidic (20:0)	0.096 (0.069–0.101)	0.057 (0.045–0.088)	0.049 (0.044–0.065)	0.135 (0.125–0.148) ^~,^*^,#^	0.125 (0.107–0.155) *^,^^	0.141 (0.106–0.163) ^~,^*
Linolenic (18n3)	0.056 (0.041–0.074)	0.045 (0.037–0.052)	0.035 (0.033–0.042)	0.066 (0.062–0.072) *^,#^	0.06 (0.051–0.066) ^	0.066 (0.058–0.08) *
Behenic (22:0)	0.205 (0.192–0.215)	0.139 (0.131–0.169) ^~^	0.137 (0.132–0.1408) ^~^	0.23 (0.215–0.239) *^,#^	0.206 (0.188–0.214) *^,^^	0.212 (0.186–0.246) *
Arachidonic (20:4n6)	0.361 (0.336–0.4)	0.521 (0.499–0.602) ^~^	0.5245 (0.487–0.556) ^~^	0.788 (0.771–0.811) ^~,^*^,#^	0.474 (0.441–0.503) ^~^	0.543 (0.513–0.61) ^~,#,^^
Lignoceric (24:0)	0.102 (0.068–0.116)	0.065 (0.055–0.126)	0.064 (0.056–0.085)	0.083 (0.06–0.107)	0.178 (0.144–0.231) ^	0.22 (0.109–0.268) ^~,^*^,^^
Eicosapentaenoic (20:5n3)	0.077 (0.064–0.1)	0.072 (0.064–0.104)	0.07 (0.066–0.082)	0.131 (0.117–0.153)	0.116 (0.098–0.166)	0.086 (0.075–0.092)
Nervonic (24:1)	0.024 (0.017–0.032)	0.021 (0.015–0.029)	0.015 (0.013–0.027)	0.037 (0.033–0.042)	0.021 (0.015–0.026)	0.016 (0.013–0.029)
Docosahexaenoic (22:6n3)	0.042 (0.04–0.065)	0.035 (0.027–0.114)	0.027 (0.021–0.046)	0.052 (0.044–0.057)	0.048 (0.043–0.152)	0.044 (0.032–0.052)
SAT	19.29 (15.094–21.256)	29.12 (28.745–32.133) ^~^	24.798 (22.665–28.939) ^~^	35.094 (33.762–36.969) ^~,^*^,#^	31.052 (30.205–32.913) ^~,^^	39.809 (38.083–41.644) ^~,^*^,#^
UNSAT	2.124 (1.733–2.325)	2.064 (1.985–2.76)	2.275 (2.094–2.586)	3.353 (3.282–3.442) ^~,^*^,#^	2.972 (2.659–3.575) ^~^	2.368 (2.191–2.877) ^
Total	21.427 (16.924–23.47)	31.184 (30.768–34.856) ^~^	27.149 (24.76–31.412) ^~^	38.385 (37.109–40.438) ^~,^*^,#^	34.275 (32.97–36.1) ^~,^^	42.177 (41.437–43.725) ^~,^*^,#^

**Table A3:** Triacylglycerols (TAG) content and composition in L6 muscle cells. The values (nmol mg^−1^ of protein) are expressed as: median (25%–75%) based on six independent determinations. The Kruskal–Wallis test followed by a post hoc adjustment was applied. ^~^ *p* < 0.05, PA versus control; * *p* < 0.05, study group (co-cultured with PQQ) versus PA; ^#^ *p* < 0.05, time-dependent differences (long- versus short-time incubation with PQQ); ^ *p* < 0.05, sequence-dependent differences (pre-incubation with PQQ versus post-incubation with PQQ). PA: palmitic acid; PQQ: pyrroloquinoline quinone; SAT: saturated fatty acids; UNSAT: unsaturated fatty acids.

TAG	Control	PA	PA/PQQ 2 h	PA/PQQ 24 h	2 h PQQ/PA	24 h PQQ/PA
Myristic (14:0)	2.847 (2.046–3.258)	5.608 (5.272–5.728) ^~^	5.608 (4.882–6.105) ^~^	11.92 (10.88–12.29) ^~,^*^,#^	5.3 (5.122–5.431) ^~^	5.976 (5.3–6.461) ^~,^^
Palmitic (16:0)	9.026 (7.286–9.664)	166.814 (156.557–174.35) ^~^	127.662 (121.291–145.259) ^~,^*	216.515 (209.856–230.962) ^~,^*^,#^	156.979 (151.511–164.094) ^~,^^	172.05 (166.321–186.678) ^~,#,^^
Palmitoleic (16:1)	0.95 (0.864–1.09)	9.394 (8.895–9.65) ^~^	12.415 (11.477–13.173) ^~,^*	18.268 (16.533–20.162) ^~,^*^,#^	10.12 (9.495–10.513) ^~,^^	6.835 (6.617–7.609) ^~,^*^,#,^^
Stearic (18:0)	2.969 (1.717–4.672)	2.35 (2.218–2.670)	2.029 (1.875–2.180)	5.022 (4.666–5.419) ^~,^*^,#,^^	3.813 (3.621–4.226) *	3.73 (3.319–3.890) *^,^^
Oleic (18:1n9c)	1.327 (1.006–1.705)	1.737 (1.614–1.954)	2.012 (1.919–2.198) ^~^	3.638 (3.364–4.211) ^~,^*^,#^	2.401 (2.239–2.750) ^~,^*	2.021 (1.784–2.299) ^~,^^
Linoleic (18:2n6c)	0.104 (0.046–0.159)	0.19 (0.184–0.262)	0.174 (0.154–0.219)	0.391 (0.349–0.45) ^~,^*^,#^	0.286 (0.255–0.341) ^~^	0.192 (0.156–0.306) ^~,^^
Arachidic (20:0)	0.104 (0.093–0.126)	0.063 (0.054–0.071) ^~^	0.056 (0.043–0.07) ^~^	0.096 (0.081–0.109) ^#^	0.09 (0.073–0.115)	0.124 (0.116–0.149) *^,#,^^
Linolenic (18n3)	0.096 (0.089–0.124)	0.184 (0.15–0.19) ^~^	0.185 (0.145–0.212) ^~^	0.264 (0.242–0.288) ^~,^*^,#^	0.211 (0.195–0.236) ^~^	0.175 (0.149–0.213) ^~,^^
Behenic (22:0)	0.159 (0.132–0.182)	0.207 (0.175–0.246)	0.207 (0.177–0.218)	0.323 (0.287–0.356) ^~,^*^,#^	0.222 (0.212–0.256) ^~^	0.243 (0.218–0.279) ^~,^^
Arachidonic (20:4n6)	0.225 (0.152–0.338)	0.383 (0.361–0.442)	0.41 (0.329–0.472) ^~^	0.52 (0.406–0.567) ^~^	0.382 (0.371–0.519) ^~^	0.432 (0.397–0.489) ^~^
Lignoceric (24:0)	0.116 (0.064–0.192)	0.095 (0.047–0.11)	0.125 (0.081–0.155)	0.071 (0.056–0.087)	0.1 (0.091–0.111)	0.142 (0.115–0.178) ^
Eicosapentaenoic (20:5n3)	0.165 (0.15–0.258)	0.182 (0.165–0.255)	0.179 (0.165–0.194)	0.277 (0.242–0.322) ^~,#^	0.209 (0.2–0.231)	0.197 (0.17–0.254)
Nervonic (24:1)	0.048 (0.03–0.058)	0.043 (0.041–0.048)	0.041 (0.036–0.043)	0.051 (0.043–0.094)	0.042 (0.033–0.055)	0.044 (0.037–0.058)
Docosahexaenoic (22:6n3)	0.091 (0.078–0.118)	0.178 (0.164–0.189)	0.206 (0.163–0.222) ^~^	0.206 (0.156–0.257) ^~^	0.226 (0.195–0.304) ^~^	0.167 (0.154–0.221)
SAT	15.353 (12.403–16.236)	175.233 (164.405–182.995) ^~^	135.633 (128.109–154.06) ^~,^*	232.864 (226.415–249.014) ^~,^*^,#^	167.144 (161.071–173.299) ^~,^^	181.373 (176.594–197.997) ^~,^^
UNSAT	3,126 (2.61–3.574)	12.278 (11.937–12.636) ^~^	15.61 (14.491–16.583) ^~,^*	23.549 (21.333–26.297) ^~,^*^,#^	13.752 (13.164–14.681) ^~^	10.029 (9.754–11.095) ^~,#,^^
Total	18.499 (15.155–19.781)	187.522 (176.396–195.565) ^~^	150.757 (143.329–171.224) ^~,^*	256.702 (247.726–275.516) ^~,^*^,#^	182.027 (173.932–187.214) ^~,^^	191.401 (186.348–209.093) ^~,^^

**Table A4:** Annealing temperature and specific primer sequences for target genes in real-time PCR reaction.

Gene	Primer Sequence		Annealing Temperature	Product Size [bp]
	Forward	Reverse		
*CD36/SR-B2*	5′-GCC TCC TTT CCA CCT TTT GT-3′	5′-GAT TCA AAC ACA GCA TAG ATG GAC-3′	56 °C	88
*FABPpm*	5′-GGA GGG TGG ATG GTG TTG AG-3′	5′-TCC AGA TAT CAG CCG TGG GA-3′	61 °C	115
*FATP1*	5′-CGC TTC TCA ATG TCA ACC TG-3′	5′-AAA TAG CCG ATC ATC CAT GC-3′	61 °C	269
*FATP4*	5′-TTG CCT GAG CTG CAC AAA AC-3′	5′-AGT GCA ACA TAG CAG CCT GT-3′	58 °C	131
*PGC-1α*	5′-TCT CGA CAC AGG TCG TGT TCC C-3’	5′-TTT CGT GCT CAT TGG CTT CAT AGC-3’	59 °C	213
*SIRT1*	5′-CAC CAG AAA GAA CTT CAC CAC CAG-3’	5′-ACC ATC AAG CCG CCT ACT AAT CTG-3’	61 °C	299
*PPARγ*	5′-ACT GGC ACC CTT GAA AAA TG-3’	5′-CCC TGG CAA AGC ATT TGT AT-3’	59 °C	201
*β-HAD*	5′-TAT CTG GGG CGG ATC ACT CT-3′	5′-CAT AGC ATG ACC CTG TCC TCC-3′	60 °C	147
*CPT1*	5′-GTC GCT TCT TCA AGG TTT GG-3′	5′-AAG AAA GCA GCA CGT TCG AT-3′	59 °C	231
*CS*	5′-GAT TGT GAA CAA TGT CCT CT-3′	5′-TTC ATC TCC GTC ATG CCA TA-3′	60 °C	108
*ATGL*	5′-CCC TGA CTC GAG TTT CGG AT-3′	5′-CAC ATA GCG CAC CCC TTG AA-3′	60 °C	145
*DGAT1*	5′-GGT TCC CTG TTT GCT CTG-3′	5′-GGC ACC ACA GAT TGA CAT-3′	58 °C	79
*DGAT2*	5′-GGA GAG TGG TAG ATA ACA-3′	5′-CAG ATT GGA GAA GAG GAG-3′	58 °C	75
*FAS*	5′-TCC CAG GTC TTG CCG TGC-3′	5′-GCG GAT GCC TAG GAT GTG TGC-3′	59 °C	259
*SPT1*	5′-CCT CAT ACT TTG GAT AAT-3′	5′-TCT TCA ATC AGT TCT TCC T-3′	58 °C	98
*GAPDH*	5′-TGC ACC ACC AAC TGC TTA-3′	5′-GGA TGC AGG GAT GAT GTT C-3′	60 °C	177
